# Chaperone-assisted E3 ligase-engineered mesenchymal stem cells target hyperglycemia-induced p53 for ubiquitination and proteasomal degradation ameliorates self-renewal

**DOI:** 10.1186/s40659-025-00604-7

**Published:** 2025-04-24

**Authors:** Ayaz Ali, Wei-Wen Kuo, Chia-Hua Kuo, Jeng-Feng Lo, Dennis Jine-Yuan Hsieh, Peiying Pai, Tsung-Jung Ho, Marthandam Asokan Shibu, Shinn-Zong Lin, Chih-Yang Huang

**Affiliations:** 1 Aberdeen Cardiovascular and Diabetes Centre, Institute of Medical Sciences, University of Aberdeen, Aberdeen, UK; 2 Department of Biological Science and Technology, China Medical University, Taichung, 404 Taiwan; 3 Laboratory of Exercise Biochemistry, University of Taipei, Taipei, Taiwan; 4 Institute of Oral Biology, National Yang-Ming University, Taipei, Taiwan; 5 School of Medical Technology, Chung Shan Medical University, Taichung, Taiwan; 6 School of Medicine, College of Medicine, China Medical University, Taichung, 40402 Taiwan; 7 Division of Cardiovascular Medicine, Department of Medicine, China Medical University Hospital, Taichung, 40447 Taiwan; 8 Department of Chinese Medicine, Hualien Tzu Chi Hospital, Buddhist Tzu Chi Medical Foundation, Tzu Chi University, Hualien, Taiwan; 9 Department of Biotechnology, Bharathiar University, Coimbatore, India; 10 Bioinnovation Center, Buddhist Tzu Chi Medical Foundation, Hualien, 970 Taiwan; 11 Department of Neurosurgery, Hualien Tzu Chi Hospital, Buddhist Tzu Chi Medical Foundation, Hualien, 970 Taiwan; 12 Graduate Institute of Biomedical Sciences, China Medical University, Taichung, 404 Taiwan; 13 Department of Medical Research, China Medical University Hospital, China Medical University, Taichung, Taiwan; 14 Department of Biotechnology, Asia University, Taichung, Taiwan; 15 Cardiovascular and Mitochondrial Related Disease Research Center, Hualien Tzu Chi Hospital, Buddhist Tzu Chi Medical Foundation, Tzu Chi University of Science and Technology, Hualien, Taiwan

**Keywords:** Carboxyl terminus of HSP70 interacting protein, p53, Hyperglycemia, Self-renewal, Mesenchymal stem cells

## Abstract

**Background:**

Stem cell therapies may potentially be used in regenerative and reconstructive medicine due to their ability for self-renewal and differentiation. Stressful conditions, such as hyperglycemia, adversely affect stem cell functions, impairing their function and promoting differentiation by opposing self-renewal. The carboxyl terminus of HSP70 interacting protein (CHIP), which is a cochaperone and E3 ligase, maintains protein homeostasis and performs quality control of the cell via ubiquitylation. However, the role of CHIP in regulating stemness remains unknown.

**Results:**

Hyperglycemia downregulated CHIP-induced p53, arrested the cell cycle at the gap (G_1_) phase, and promoted the loss of stemness in WJMSCs. Quantitative real-time polymerase chain reaction (qRT-PCR), Western blotting, immunofluorescence, and cell cycle analysis showed that CHIP-overexpressing WJMSCs downregulated the expression of phosphorylated p53 and shortened its half-life while enhancing self-renewal factors. Additionally, co-IP and Western blotting revealed that CHIP promoted the ubiquitination and proteasomal degradation of hyperglycemia-induced p53 through the chaperone system.

**Conclusions:**

CHIP may promote ubiquitin-mediated proteasomal degradation of hyperglycemia-induced p53 rescues self-renewal genes, which can maintain the long-term undifferentiated state of WJMSCs. CHIP may be an alternative therapeutic option in regenerative medicine for hyperglycemic-related complications in diabetes.

**Supplementary Information:**

The online version contains supplementary material available at 10.1186/s40659-025-00604-7.

## Introduction

Diabetes is characterized by elevated glucose levels during type 1 diabetes. Stolzing et al. revealed that hyperglycemia negatively affected the proliferation, viability, colony formation, and differentiation of bone marrow mesenchymal stem cells (MSC) [1, 2]. Self-renewal is the process by which stem cells divide to proliferate and increase the stem cell pool throughout life. This process maintains the undifferentiated state, allowing for self-renewal and multipotential differentiation [3, 4]. MSCs are used in tissue engineering due to their high rate of self-renewal and ability to differentiate into chondrocytes, osteoblasts, adipocytes, cardiomyocytes, and hepatocytes as well as ectodermal and endodermal cells [5, 6]. Keats and Khan investigated whether hyperglycemia affects activation and induces dysfunction in SCs, revealing that although endothelial progenitor cells are resistant to hyperglycemia, reduced growth and altered differentiation potential may occur [7]. Phadnis et al. evaluated the diabetic microenvironment surrounding bone marrow-derived MSCs isolated from persons with diabetes and revealed that hyperglycemia can induce the differentiation of human SCs [8]. Thus, hyperglycemia can adversely affect SC functionality, warranting strategies to overcome this problem.

MSCs isolated from several sites, including Wharton’s jelly [9], umbilical cord [10], and adipose tissues, repair damaged structures, modulate immunity, and possess stemness [11]. Wharton’s jelly-derived MSCs (WJMSCs) are a fibroblast-like homogenous population of SCs that are distinct from other SCs [12, 13]. Characteristics unique to WJMSCs include stemness maintenance, therapeutic capability, immune privileged, ease of isolation, and multi-lineage differentiation potential, making them an ideal therapeutic agent in regenerative medicine [14, 15]. However, there are few studies regarding the stemness of WJMSCs in hyperglycemic conditions.

P53, which is a tumor suppressor protein, regulates self-renewal, differentiation, cell cycle progression, apoptosis, and senescence; therefore, it should be strictly regulated [16, 17]. Normally, p53 activity is maintained at low levels, but stress upregulates it [16]. Additionally, hyperglycemic conditions may also upregulate p53 phosphorylation, leading to cellular dysfunction [18]. P53 also promotes the differentiation of various SC lines, including embryonic and neural SCs, by opposing self-renewal [17, 19]. Several E3 ligases are reportedly involved in regulating p53 activity, including MDM2 and COP1 [16]. Notably, the carboxyl terminus of the HSP70 interacting protein (CHIP), which is an E3 ligase and a cochaperone for HSP70 and HSP90, can also regulate p53 activity; however, its impact on stemness in hyperglycemic conditions remains unknown.

The ubiquitin–proteasome system plays an important role in protein homeostasis by catalyzing the destruction of misfolded or impaired cellular proteins in stem cells [20]. Several E3 ligase components, such as Itch, Skp1, and Wwp1, regulate self-renewal, cell binding, and differentiation [21, 22]. Additionally, NANOG and c-Myc are responsible for maintaining pluripotency in embryonic SCs and are regulated through ubiquitylation [23]. CHIP is a highly conserved protein among species such as mice and fruit flies [24]. CHIP contains two domains: a U-box domain located at the C terminus that possesses the E3 ligase activity and a tetratricopeptide repeat (TRP) domain positioned at the N terminus that interacts with the chaperone (HSP70/90) substrates [25, 26]. We have established the protective role of CHIP-overexpressing WJMSCs against diabetes-induced cardiac and kidney injuries in vitro and in vivo [27, 28]. Furthermore, CHIP is protective against doxorubicin-mediated cardiac cell death [29]. Moreover, CHIP can target p53 for ubiquitin-mediated proteasomal degradation [30], suggesting that CHIP can regulate several cellular functions that are normally performed by other proteins.

Hyperglycemia adversely affects SC functions by inducing survival resistance, senescence, and differentiation, preventing SCs from maintaining an undifferentiated state. Therefore, we hypothesized that during hyperglycemia, CHIP activity is downregulated, and it is unable to perform its function, resulting in upregulation of p53 activity and leading to the loss of stemness of WJMSCs. Overexpressing CHIP, a chaperone-assisted E3 ligase might augment WJMSCs self-renewal potential by targeting hyperglycemia induced p53 for ubiquitin-mediated proteasomal degradation and can be used as an alternate therapeutic strategy in regenerative medicine against diabetes-induced complications.

## Materials and methods

### Reagents, plasmids, and antibodies

All the small interfering RNAs (siRNAs) and chemicals were purchased from Sigma Aldrich (St. Louis, USA) unless and otherwise stated. HSP90 inhibitor geldanamycin (GA) was procured from Biosciences.

The PRK5 plasmid backbone encoding CHIP coding sequence was provided by Dr. Jeng-Fan Lo (National Yang‐Ming Medical University, Taipei, Taiwan). Lentiviral plasmids expressing small hairpin RNAs (shRNAs), including shcontrol (pLAS.Void), shCHIP (TRCN0000007528 NM_005861), and shp53 (TRCN0000003756NM_000546) were obtained from the national RNAi core facility (academia sinica, Taipei, Taiwan).

The primary antibodies used in this study are: rabbit anti-CHIP (H-231) (sc-66830), mouse anti-HA (F-7) (sc-7392), mouse anti-ubiquitin (P4D1) (sc-8017), mouse anti-β-actin (C4) (sc-47778), (Santa Cruz Biotechnology, CA, USA), mouse anti-p53 (1C12) (#2524), rabbit anti-P-p53^Ser15^ (#9284), mouse anti-Sox2 (L1D6A2) (#4900), rabbit anti-Oct4 (#2750), and rabbit anti-Nanog (D73G4) XP (#4903) (Cell Signaling Technology, MA, USA). The secondary antibodies conjugated with horseradish peroxidase were procured from Santa Cruz Biotechnology (CA, USA).

### Cell culture and transient transfection/gene silencing

WJMSCs obtained from Bioresource Collection and Research Center (BCRC, Taipei, Taiwan) maintained in 5% CO_2_ incubator at 37 °C were used at the fourth passage to exclude aging effects. In brief, cells grown in Dulbecco’s Modified Eagle’s Medium (DMEM) provided with 10% fetal bovine serum (HyClone, CA, USA), 100 U/ml penicillin, 2 mM glutamine, 1.5 g/L sodium bicarbonate, and 100 mg/ml streptomycin were incubated with hyperglycemia and thereafter transfected with plasmids and/or shRNAs following the manufacturer guidelines. MG-132, a proteasome inhibitor and cycloheximide (CHX), a protein synthesis inhibitor were treated after cells were challenged with hyperglycemia.

### Western blotting

Western blotting was executed as delineated in previous studies [31, 32]. Briefly, WJMSCs were lysed (50 mM Tris-base, 1 M EDTA, 0.5 M NaCl, 1 mM beta-mercaptoethanol, 1% NP-40, protease inhibitor tablet (Roche, Manheim, Germany)) and centrifuged at 10,000 × *g* for 30 min followed by quantification using Bradford assay. Then, the whole cell lysate (30–40 µg) was separated by sodium dodecyl sulfate polyacrylamide gel electrophoresis (SDS-PAGE) (10–12%) and transferred to a polyvinylidene fluoride (PVDF) membrane using a constant voltage of 80–100 V followed by blocking for 1 h at room temperature using 5% skimmed milk. The membranes were probed with the respective primary antibodies (1:1000) overnight at 4 °C followed by secondary antibodies (1:3000) incubation for 1 h. Finally, the membranes were scanned with LAS 3000 imaging system (Fujifilm, Tokyo, Japan), and photomicrographs were further processed and analyzed using ImageJ and GraphPad Prism8 software.

### Quantitative real time polymerase chain reaction (qRT-PCR)

Total cellular RNA was extracted and purified from WJMSCs using RNA miniprep kit (Zymo Research Corporation, Irvine, CA, USA) following manufacturer instruction. Reverse transcription was performed and synthesized cDNA using iScript synthesis kit (Bio-Rad, CA, USA) was amplified for qRT-PCR with Fast SYBR™ Green Master Mix (Applied Biosystems, Thermo Fisher Scientific) on a QuantStudio 5 (Applied Biosystems, Thermo Fisher Scientific) with initial denaturation (95 °C for 10 min), 40 cycles of amplification (denaturation at 95 °C for 30 s; amplification at 55 °C for 1 min; and extension at 72 °C for 30 s). All the reactions were performed in triplicate and the primers used in this study were listed in Table S1. The threshold cycle (Ct) value for each gene was determined and the values were normalized to GAPDH using the 2^ΔCt^ method.

### Immunofluorescence

WJMSCs cultured in chamber slides (SPL Life Sciences, Korea) were fixed with 4% paraformaldehyde for 30 min, permeabilized using 0.1% Triton X-100 for 10 min, and subsequently blocked in 1% BSA for 2 h to avoid the nonspecific binding. Thereafter, cells were probed with the anti-p53 (1:500) primary antibody at 4 °C overnight followed by incubation with Alexa Fluor 488 goat anti-mouse IgG (1:200) secondary antibody (Invitrogen). Cells were stained with DAPI for 5 min to examine the nucleus, and photomicrographs were acquired and processed.

### Co-Immunoprecipitation (Co-IP)

Co-IP was performed as delineated in our recent study to analyze the interaction between CHIP and p53 [27]. Total cellular extract obtained from the WJMSCs were immunoprecipitated using Protein G magnetic beads (Millipore) according to manufacturer instructions. Primary antibody (2 µg) was incubated with the total cellular extract (500 µg) on a rotator overnight at 4 °C. Immunoprecipitated cellular proteins were washed, eluted using magnetic rack and separated on SDS-PAGE followed by transfer onto PVDF membrane following the above-described western blotting method.

### Cell cycle analysis

Cell cycle was performed to analyze the different phases of cell. Firstly, WJMSCs challenged with high glucose (HG) and transfected with different plasmids, including HA-CHIP, shCHIP, and shP53 were harvested followed by washing with cold PBS twice. Then, cells were fixed on a rotor with ice chilled 70% ethanol and kept at 4 °C overnight. Thereafter, cells were washed three times with PBS and incubated with propidium iodide (1 mg/ml) solution containing RNase A (0.4 mg/ml) for 15 min at room temperature. The DNA content was analyzed using FACS Canto™ flow cytometry. The cell cycle events were based on *n* = 10,000 cells.

### Statistical analysis

Data are shown as ± SD. Statistical analysis was performed using ImageJ and GraphPad Prism8 software. The Shapiro-Wilk test was performed to assess the normal distribution and then multiple comparisons of one or two independent variables were assessed by one way and two-way analysis of variance respectively followed by Tukey’s or Bonferroni post-hoc test. P value of < 0.05 represents statistical significance.

## Results

### Hyperglycemia-impaired CHIP-stabilized p53 and promoted the loss of stemness in WJMSCs

**Fig. 1:**
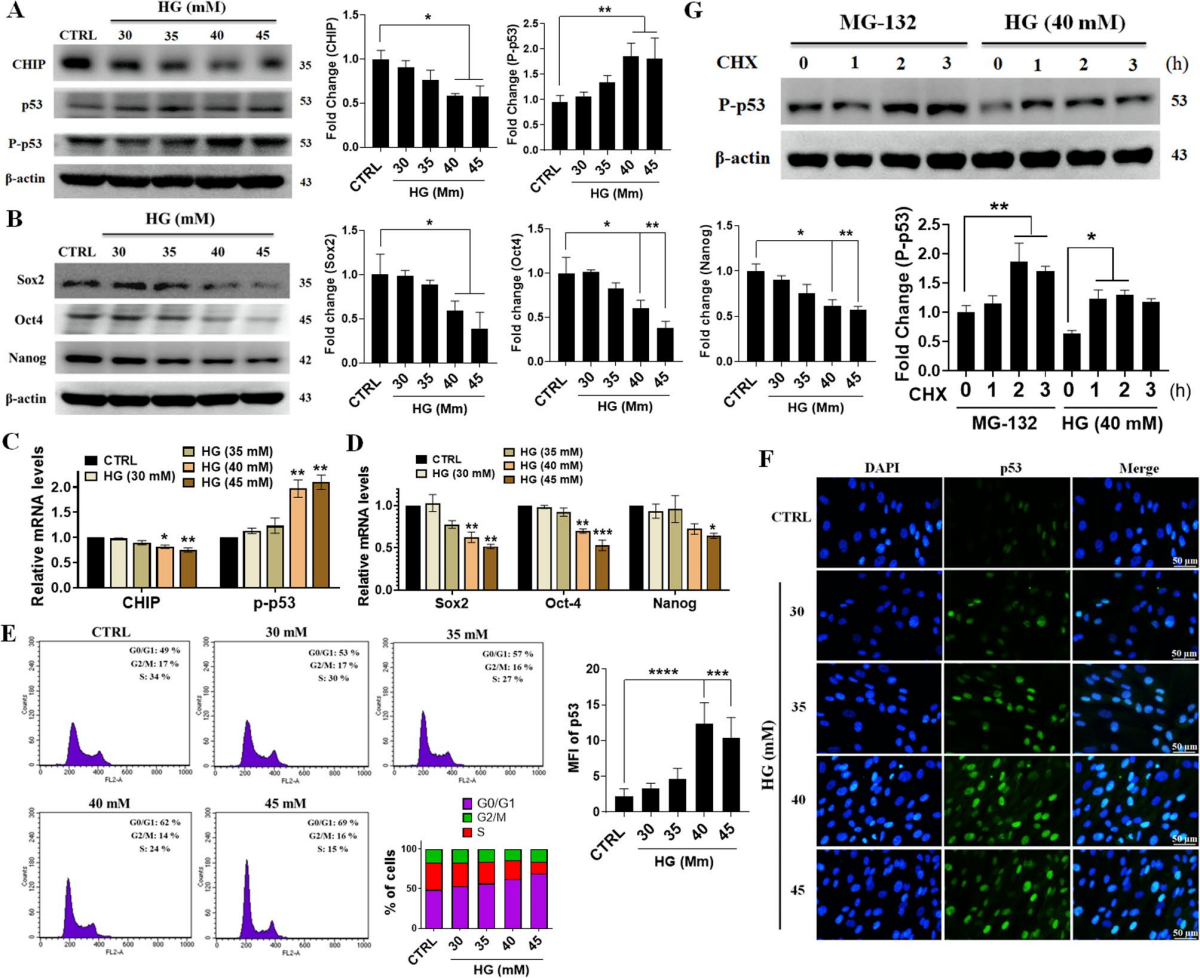
Hyperglycemia impaired CHIP stabilized P53 promotes the loss of self-renewal in WJMSCs. (**A** and **B**) WJMSCs cultured under increasing concentrations of HG (30, 35, 40, and 45 mM) for 3d were harvested and the expression levels of CHIP, p53 and self-renewal genes (SOX2, OCT-4, and NANOG) were analyzed using western blot analysis. (**C** and **D**) Cells seeded under increasing concentrations of HG for 3d were harvested and the mRNA levels of CHIP, p53, and self-renewal genes were analyzed using qRT-PCR. (**E**) WJMSCs cultured under increasing concentrations of HG for 3d and the cell cycle was determined using FACS analysis. (**F**) WJMSCs seeded for 3d with indicated concentrations of HG were stained with p53 and expression was measured using fluorescence microscopy. (**G**) Cells incubated with either MG-132 (proteasome inhibitor) or HG for different time points (0, 1, 2, 3 h) in the presence of cycloheximide (CHX) (protein synthesis inhibitor) for 6 h were immunoblotted using anti-p53 antibody. β-actin used as internal loading control and *p < 0.05, **p < 0.01, ***p < 0.001, and ****p < 0.0001 shows the significant difference (*n* = 3 per group). Scale bar indicates 50 μm

Previous studies have demonstrated that p53 induces the loss of self-renewal in several cell lines, including neural and embryonic stem cells [17, 19]. Therefore, we evaluated the effect of HG on CHIP, p53, and self-renewal factors in WJMSCs. Western blotting revealed that hyperglycemia-suppressed CHIP induces p53 expression, resulting in the loss of self-renewal factors, such as Sox2, Oct4, and Nanog, in a dose-dependent manner (Fig. 1A-B). Similarly, hyperglycemia significantly decreased mRNA levels of CHIP, while p53 expression increased in a dose-dependent manner (Fig. 1C). qRT-PCR revealed that Sox2, Oct-4, and Nanog levels were downregulated during hyperglycemic (40 and 45 mM) conditions (Fig. 1D). During loss of self-renewal, SCs stay in G1 and exhibit a lengthened cell cycle. Flow cytometry analysis revealed that during hyperglycemia, the cell cycle of WJMSCs was arrested at G1, indicating loss of stemness in a dose-dependent manner (Fig. 1E). Additionally, immunofluorescence staining showed increased p53 activity in 40 and 45 mM of glucose; thereafter, 40 mM of glucose was chosen as the optimal dose for further experiments, which can promote the loss of self-renewal and induce p53 expression (Fig. 1F). To determine whether hyperglycemia can stabilize p53 expression, Western blotting was performed, revealing that treatment with cycloheximide (CHX) protein synthesis inhibitor) in the presence of either MG-132 (proteasome-mediated degradation inhibitor) or hyperglycemia-stabilized phosphorylated p53 due to its inefficient degradation (Fig. 1G). These results suggest that hyperglycemia-stabilized phosphorylated p53 is associated with the loss of stemness caused by CHIP impairment in WJMSCs.

### CHIP overexpression maintained self-renewal factors by suppressing hyperglycemia-induced p53

**Fig. 2:**
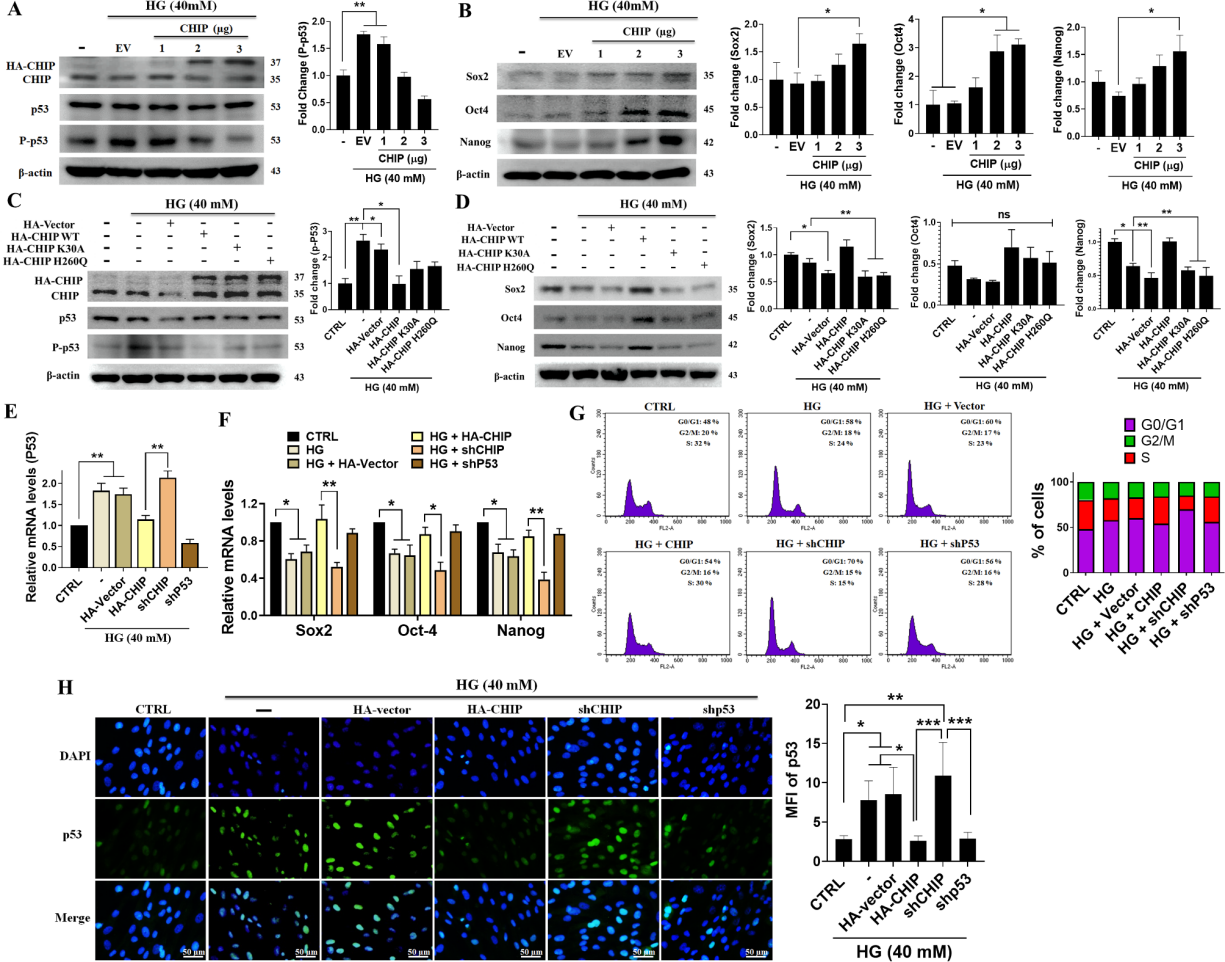
CHIP overexpression attenuated hyperglycemia-induced p53 enhanced self-renewal in WJMSCs. (**A** and **B**) Cells transfected with increasing concentrations of CHIP (1, 2, 3 µg) were challenged with HG for 3 d followed by western blot analysis to measure the protein expression of CHIP, phosphorylated p53, and the self-renewal genes (SOX2, OCT-4, and NANOG). (**C** and **D**) WJMSCs transfected with either HA-vector, HA-CHIP, or HA-CHIP mutants (K30A and H260Q) under HG conditions for 3 d were harvested and the protein expression level was measured using western blot analysis. (**E and F**) Cells transfected with HA-vector, HA-CHIP, lentiviral shCHIP, and shp53 plasmids in the presence of HG for 3 d were subjected to qRT-PCR to measure the expression of p53 and self-renewal genes. (**G**) WJMSCs transfected with HA-vector, HA-CHIP, shCHIP, and shP53 plasmids after challenged with HG for 3 d were subjected to analyze cell cycle. (**H**) WJMSCs transfected with either HA-vector, HA-CHIP, shCHIP, and/or shp53 plasmids were incubated with HG for 3 d and the expression level of p53 was assessed using fluorescence microscopy. β-actin served as loading control and *p < 0.05, **p < 0.01, and ***p < 0.001 indicates the significant difference (*n* = 3 per group). Scale bar represents 50 μm

Our previous study showed that CHIP could enhance the stemness properties of WJMSCs [33]. Considering this, we evaluated whether CHIP can rescue the WJMSC self-renewal ability in the HG challenge. Western blotting revealed that CHIP overexpression downregulated hyperglycemia-activated p53, whereas the expression of Sox2, Oct-4, and Nanog were upregulated in a dose-dependent manner (Fig. 2A-B). We also confirmed the role of CHIP by employing the CHIP mutants K30A (N-terminal domain mutant) and H260Q (C-terminal domain mutant). Immunoblotting revealed that overexpression of wild-type CHIP suppressed hyperglycemia-activated p53, thereby inducing Sox2, Oct-4, and Nanog; however, the empty vector and neither of the CHIP mutants produced the desired effect (Fig. 2C-D). qRT-PCR was performed to analyze the impact of CHIP on p53 and self-renewal genes, revealing that the p53 transcript level was significantly increased in WJMSCs carrying the HA-vector and shCHIP plasmids; however, these effects were reversed upon CHIP overexpression and p53 knockdown (Fig. 2E). Moreover, the expression of Sox2, Oct-4, and Nanog, which was downregulated in WJMSCs carrying the HA-vector and shCHIP due to hyperglycemia, reverted to normal following CHIP overexpression and p53 knockdown (Fig. 2F). Furthermore, CHIP overexpression and p53 knockdown inhibited the cell cycle arrest at G1 phase (Fig. 2G). Similarly, immunofluorescence revealed that hyperglycemia-induced p53 activity was downregulated in the CHIP-overexpressing and p53 knockdown groups as compared to the vector and CHIP knockdown groups (Fig. 2H). These indicate that CHIP overexpression suppressed hyperglycemia-induced p53 phosphorylation, thereby enhancing the expression of Sox2, Oct-4, and Nanog. Nevertheless, both CHIP mutants were unable to produce any effect under hyperglycemic conditions.

### CHIP regulates hyperglycemia-induced p53 and decreases its half-life in WJMSCs

**Fig. 3:**
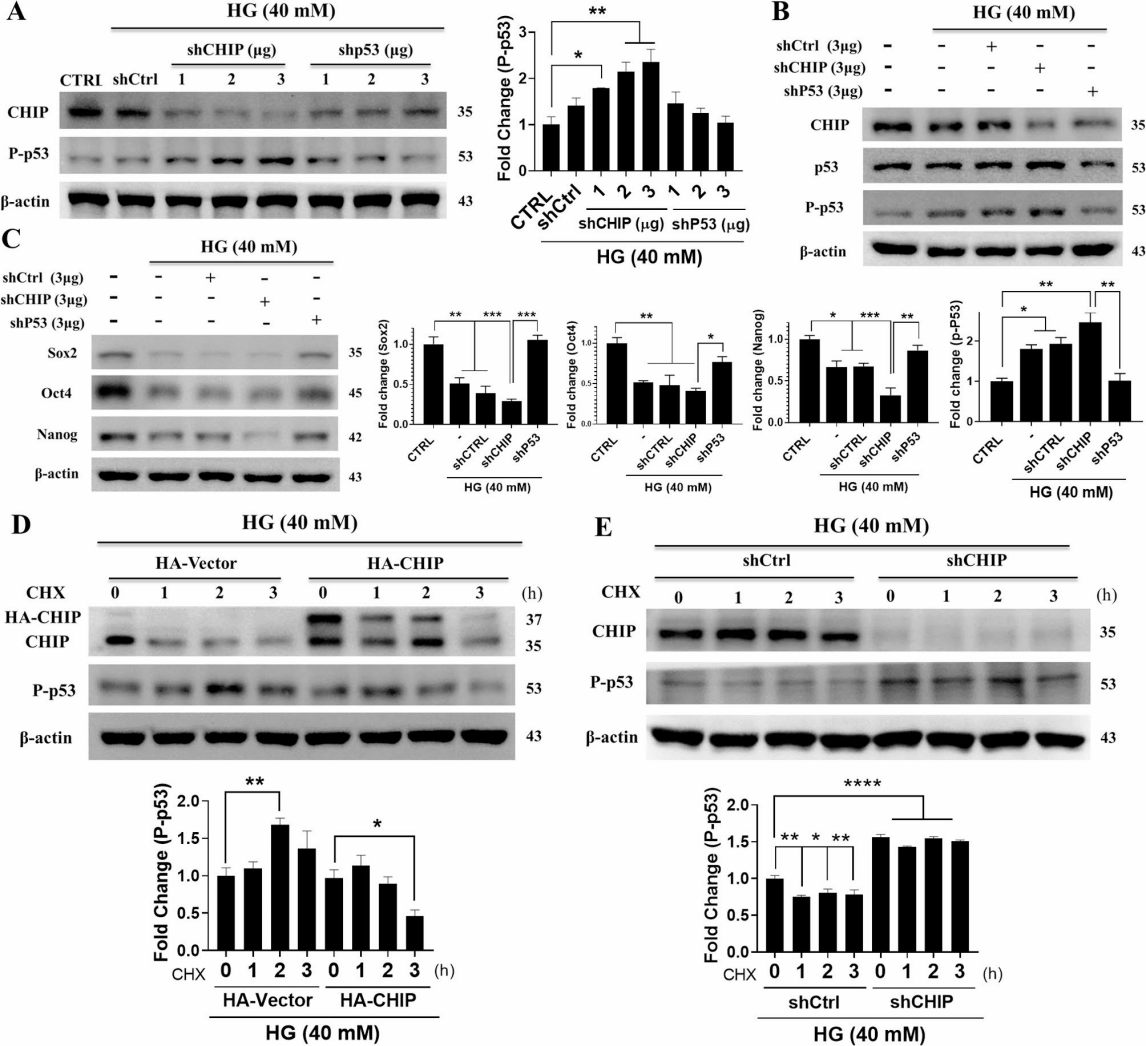
CHIP regulates p53 half-life under HG conditions in WJMSCs. (**A**) Cells transfected with either shCHIP or shp53 plasmids in the presence of HG were subjected to western blot analysis and the expression level of CHIP and p53 was measured. (**B** and **C**) WJMSCs transfected with shcontrol, shCHIP, and shp53 plasmids were challenged with HG followed by immunoblotting to measure the expression level of CHIP, phosphorylated p53, and self-renewal genes (SOX2, OCT-4, and NANOG). (**D** and **E**) WJMSCs transfected with HA-CHIP and lentiviral shCHIP after challenged with HG in the presence of CHX (50 µg/ml) for the indicated time points (1, 2, 3 h) were subjected to western blot analysis, and the protein expression of CHIP and phosphorylated p53 was measured. β-actin used as internal control. *p < 0.05, **p < 0.01, ***p < 0.001, and ****p < 0.0001 represents the significance (*n* = 3 per group)

We also evaluated the effect of CHIP knockdown on p53 under hyperglycemic conditions in WJMSCs. Western blotting revealed that silencing CHIP upregulated phosphorylated p53 compared with the control and hyperglycemia alone groups; however, its expression was downregulated by p53 inhibition (Fig. 3A-B). We then evaluated the impact of CHIP knockdown on the factors responsible for maintaining WJMSC stemness. We noted that the activity of self-renewal factors, which was already downregulated during hyperglycemia, was further inhibited following CHIP silencing but rescued by p53 knockdown (Fig. 3C). Moreover, we determined the influence of CHIP by gain and loss of function on endogenous phosphorylated p53 levels after treatment with CHX (protein synthesis inhibitor) during hyperglycemia. Immunoblotting revealed that the half-life of hyperglycemia-activated p53 was either decreased or increased following CHIP overexpression and knockdown, respectively (Fig. 3D-E). These results suggest that CHIP regulates hyperglycemia-activated p53 and its half-life in WJMSCs.

### CHIP promotes the ubiquitin-mediated proteasomal degradation of hyperglycemia-induced p53 in WJMSCs

**Fig. 4:**
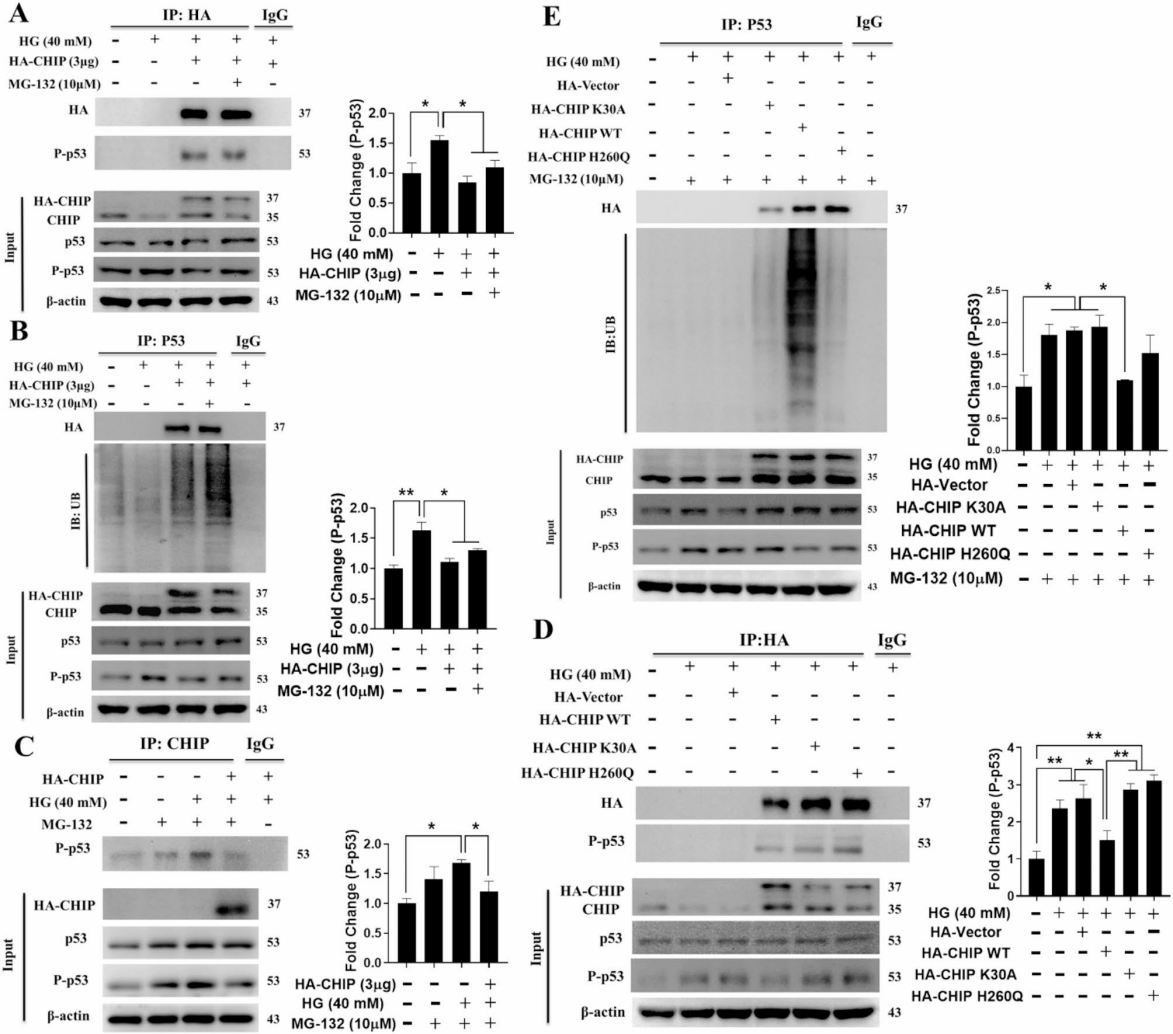
CHIP targets HG-induced p53 for ubiquitin-mediated proteasomal degradation in WJMSCs. (**A**-**C**) WJMSCs transfected with HA-CHIP plasmid in the presence of MG-132 for 6 h were challenged with HG for 3 d. Total cellular lysate immunoprecipitated with the anti-HA, anti-P-p53, and anti-CHIP antibodies were further immunoblotted with anti-HA, anti-P-p53, and anti-ubiquitin antibody. (**D** and **E**) WJMSCs transfected with HA-vector, HA-CHIP, and CHIP mutants (K30A and H260Q) were challenged with HG in the absence and presence of MG-132 for 6 h. Total cellular lysate immunoprecipitated with the anti-HA and anti-p53 antibody was subsequently immunoblotted with the anti-HA, anti-P-p53, and anti-ubiquitin antibody. β-actin employed as internal loading control and *p < 0.05, and **p < 0.01 indicates the significance (*n* = 3 per group)

We then explored whether CHIP can directly ubiquitinate p53 for proteasomal degradation during hyperglycemia. Data from the co-IP assay indicated that CHIP overexpression suppressed hyperglycemia-induced p53 expression (Fig. 4A), directly interacted with phosphorylated p53 for ubiquitination, allowing for ubiquitination-mediated proteasomal degradation (Fig. 4B-C). Further, co-IP assay was then performed to analyze the binding as well as the E3 ligase ability of CHIP during hyperglycemia. Results showed that only the wild-type CHIP promotes ubiquitin-mediated proteasomal degradation of phosphorylated p53. Although the CHIP mutants (K30A and H260Q) can bind with p53, neither of them can induce ubiquitination, suggesting that both domains of CHIP are involved in ubiquitination and proteasomal degradation of hyperglycemia-activated p53, and possibly the chaperone system is involved in assisting ubiquitination of p53 as K30A domain mutant lose its potential to interact with chaperones (HSP70/90) (Fig. 4D-E). These results indicate that CHIP can interact directly with hyperglycemia-activated p53 and promote its ubiquitination and proteasomal degradation in WJMSCs.

### The chaperone system is involved in CHIP-mediated proteasomal degradation of p53 during hyperglycemia in WJMSCs

**Fig. 5:**
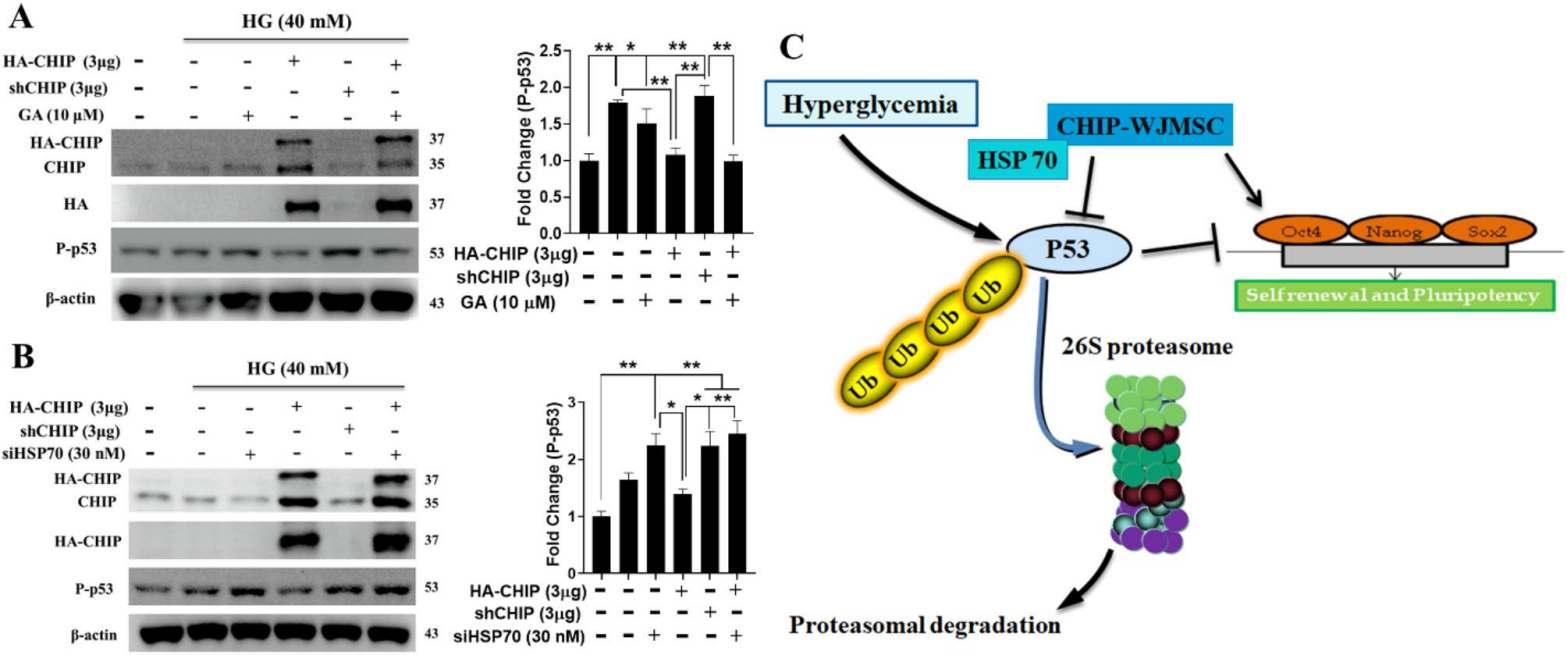
Chaperone system is involved in proteasomal degradation of HG-induced p53 in WJMSCs. (**A**) WJMSCs transfected with either HA-CHIP, shCHIP or treated with GA (HSP90 inhibitor) were challenged with the HG and total cellular lysate was analyzed to measure the protein expression of CHIP, HA and phosphorylated p53 using western blot analysis. (**B**) WJMSCs transfected with HA-CHIP, shCHIP, and siHSP70 were incubated with HG and immunoblotted to measure the expression level of CHIP, HA-tagged CHIP, and phosphorylated p53. (**C**) Graphical representation illustrating that CHIP targets HG-induced p53 for ubiquitination-mediated proteasomal degradation ameliorates self-renewal factors. β-actin employed as internal loading control and *p < 0.05, and **p < 0.01 indicates the significance (*n* = 3 per group)

We then evaluated the involvement of HSP70/90 in CHIP-mediated proteasomal degradation of p53. Western blotting revealed that the activities of CHIP alone and together with GA (HSP90 inhibitor) correlated with the suppression of hyperglycemia-induced p53, while CHIP depletion was unable to produce the desired effect (Fig. 5A). We then assessed the effect of HSP70 on CHIP-mediated degradation of hyperglycemia-induced p53 as CHIP regulates several proteins presented by HSP70. Results showed that the CHIP-induced downregulation of hyperglycemia-induced p53 was blocked by HSP70 and CHIP knockdown in the absence and presence of CHIP during hyperglycemia, respectively, suggesting the cooperation of HSP70 in CHIP-mediated p53 degradation (Fig. 5B). The results suggest that CHIP can maintain the stemness of WJMSCs during hyperglycemia by promoting the ubiquitination and proteasomal degradation of phosphorylated p53 through the chaperone system (Fig. 5C).

## Discussion

During stressful conditions, SCs may lose their therapeutic and regenerative properties. A previous study demonstrated the adverse effects of hyperglycemia on various SC lines including bone-marrow MSCs, muscle-derived MSCs, placenta-derived MSCs [34]. Additionally, a study showed that p53 expression increases during hyperglycemia [18]. Furthermore, stress-activated p53 is caused by the post-translational modification and protein stability of p53 [35]. Moreover, activated p53 prolongs the G1 phase and lengthens the cell cycle, leading to the differentiation of human embryonic SCs with minimal effect in inducing apoptosis [17]. Therefore, it would be beneficial to augment WJMSC robustness by possibly using CHIP to enhance their stemness in hyperglycemic conditions. Our results showed that hyperglycemia reduced the functional activity of CHIP-stabilized phosphorylated p53, and that there was an increase in cell cycle arrest at G1 phase, indicating the loss of stemness that leads to the inability of stem cells to maintain their long-term undifferentiated state.

P53 negatively regulates the self-renewal capacity by promoting differentiation in various stem cell lines such as neural and embryonic stem cells [17, 19]. The role of p53 modulation was first documented by repressing Nanog in mouse embryonic stem cells [36]. P53 regulates apoptosis and differentiation of stem cells; however, the mechanistic insights responsible for regulating p53 under hyperglycemic conditions and its impact on self-renewal potential in WJMSCs remain unknown. Proteasomal degradation mediated by ubiquitination plays an important role in maintaining the protein quality that maintains cellular homeostasis [37]. Additionally, CHIP may promote the ubiquitination and proteasomal degradation of various proteins [38, 39] and can target p53 for proteasomal degradation. Several proteins with E3 ligase function can regulate the functionality and stability of various protein factors such as c-Myc and Oct-4 in adult-derived multipotent MSCs and embryonic stem cells, suggesting that the ubiquitylation pathway maintains the balance among self-renewal, vesicle trafficking, differentiation, and quiescence [40]. Overall, our findings suggest that CHIP may target hyperglycemia-activated p53 for ubiquitin-mediated proteasomal degradation through chaperone systems such as HSP90 and HSP70; this is aligned with previous studies. Our findings increased the current understanding of the role of CHIP in enhancing the self-renewal capacity to maintain stemness by regulating phosphorylated p53 in hyperglycemic conditions.

Diabetes is characterized by hyperglycemia, which can adversely affect stem cell functionality. Therefore, novel therapies are needed to overcome the adverse outcomes caused by hyperglycemia, such as loss of stemness. Engineered stem cells are efficacious against several disease conditions [41, 42]. In fact, stem cell-based therapeutics have gained much attention among the research community as genetic engineering may enhance the therapeutic potential of stem cells. Considering this, our CHIP-carrying WJMSCs can be employed as potential therapeutic agents during hyperglycemic conditions.

The molecular interactions responsible for the hyperglycemia-induced p53-mediated loss of self-renewal and CHIP overexpression in WJMSCs provides evidence that self-renewal factors enhance the stemness potential during hyperglycemia. We believe that this finding contributes to the current literature and reveals the potential of CHIP-overexpressing WJMSCs to be used in regenerative medicine. A possible limitation of this study is that the effect of CHIP-carrying MSCs was only observed in vitro, and in vivo data to support to test its efficacy and effectiveness as a therapeutic strategy in clinical treatment against diabetes-induced damages is lacking. Further studies are required to elucidate the differentiation capacity of CHIP-overexpressing SCs.

## Conclusion

Hyperglycemia promotes the loss of stemness of WJMSCs. CHIP, a co-chaperone and E3 ubiquitin ligase maintains cellular homeostasis by mediating various substrates for proteasomal degradation. During hyperglycemic condition, CHIP lost its function stabilized p53 and WJMSCs lost its self-renewal function and thereby unable to maintain their long-term undifferentiated state. CHIP overexpression downregulates p53 and promotes its ubiquitination mediated by proteasomal degradation rescues the self-renewal factors through the involvement of the chaperone system. This study highlights the use of CHIP-carrying WJMSCs as a therapeutic agent in regenerative medicine against hyperglycemia-induced complications during diabetes.

## Electronic supplementary material

Below is the link to the electronic supplementary material.

Supplementary Material 1

Supplementary Material 2

## Data Availability

All data generated or analyzed during this study are included in this article.

## Abbreviations

CHIP: Carboxyl-Terminus of Heat shock protein 70-Interacting Protein
DAPI: 4`, 6-Diamidino-2-Phenylindole
CHX: Cycloheximide
Co-IP: Co-Immunoprecipitation
G1: Gap1
GA: Geldanamycin
HA: Hemagglutinin
HG: High Glucose
qRT-PCR: Quantitative Real Time Polymerase Chain Reaction
shRNAs: small hairpin Robonucleic Acids
siRNAs: small interfering Ribonucleic Acids
WJMSCs: Wharton’s Jelly Derived Mesenchymal Stem Cells
