# Supplementary material for: Chaperone-assisted E3 ligase-engineered mesenchymal stem cells target hyperglycemia-induced p53 for ubiquitination and proteasomal degradation ameliorates self-renewal
Source: Biol Res. 2025 Apr 24;58:20. doi: 10.1186/s40659-025-00604-7 (PMC12020092; doi:10.1186/s40659-025-00604-7)
Supplement: Supplementary file 1 — Supplementary Material 1 [file 40659_2025_604_MOESM1_ESM.docx]

Table S1: Real Time qRT-PCR Primers list

| **Gene** | **Primers** |
| --- | --- |
| hCHIP-5' | CCTATGACCGCAAGGACATT |
| hCHIP-3' | CTCTACCCAGCCGTTCTCAG |
| hTP53-5’ | GCGAGCACTGCCCAACAACA |
| hTP53-3’ | GGATCTGAAGGGTGAAATATTCT |
| GAPDH-5’ | GCACCGTCAAGGCTGAGAAC |
| GAPDH-3’ | ATGGTGGTGAAGACGCCAGT |
| hOCT4-5' | ACATCAAAGCTCTGCAGAAAGAACT |
| hOCT4-3' | CTGAATACCTTCCCAAATAGAACCC |
| hNANOG-5’ | CCGAAGAATAGCAATGGTGTGACG |
| hNANOG-3’ | AGGAGAATTTGGCTGGAACTGC |
| hSOX2-5' | CGCCGCCCCCAGCAGACTTCACAT |
| hSOX2-3' | TGCACCCCTCCCATTTCCCTCGTT |
