# Supplementary material for: Chaperone-assisted E3 ligase-engineered mesenchymal stem cells target hyperglycemia-induced p53 for ubiquitination and proteasomal degradation ameliorates self-renewal
Source: Biol Res. 2025 Apr 24;58:20. doi: 10.1186/s40659-025-00604-7 (PMC12020092; doi:10.1186/s40659-025-00604-7)
Supplement: Supplementary file 2 — Supplementary Material 2 [file 40659_2025_604_MOESM2_ESM.pdf]

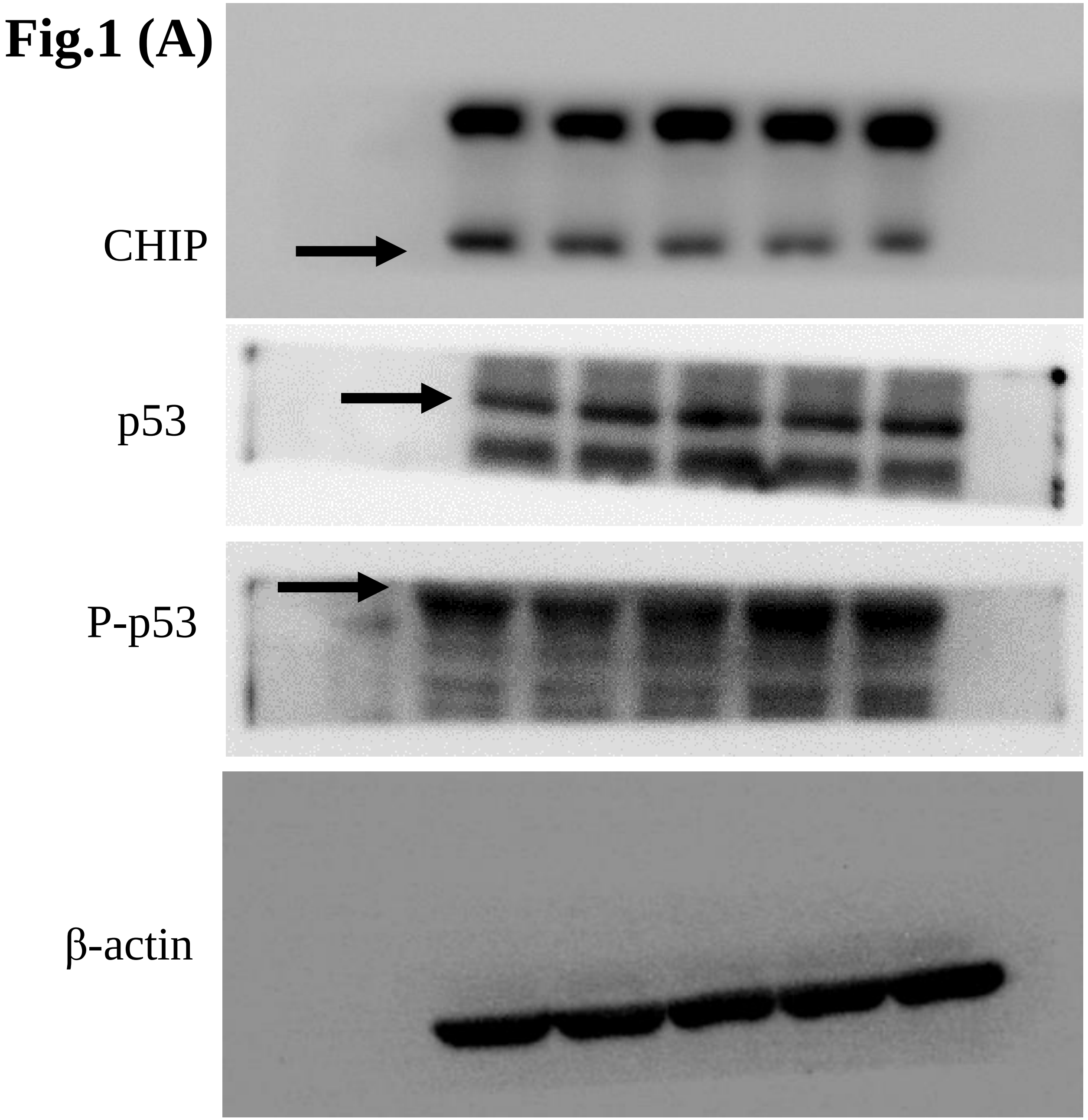
Fig.1 (A)
CHIP
p53
P-p53
β-actin

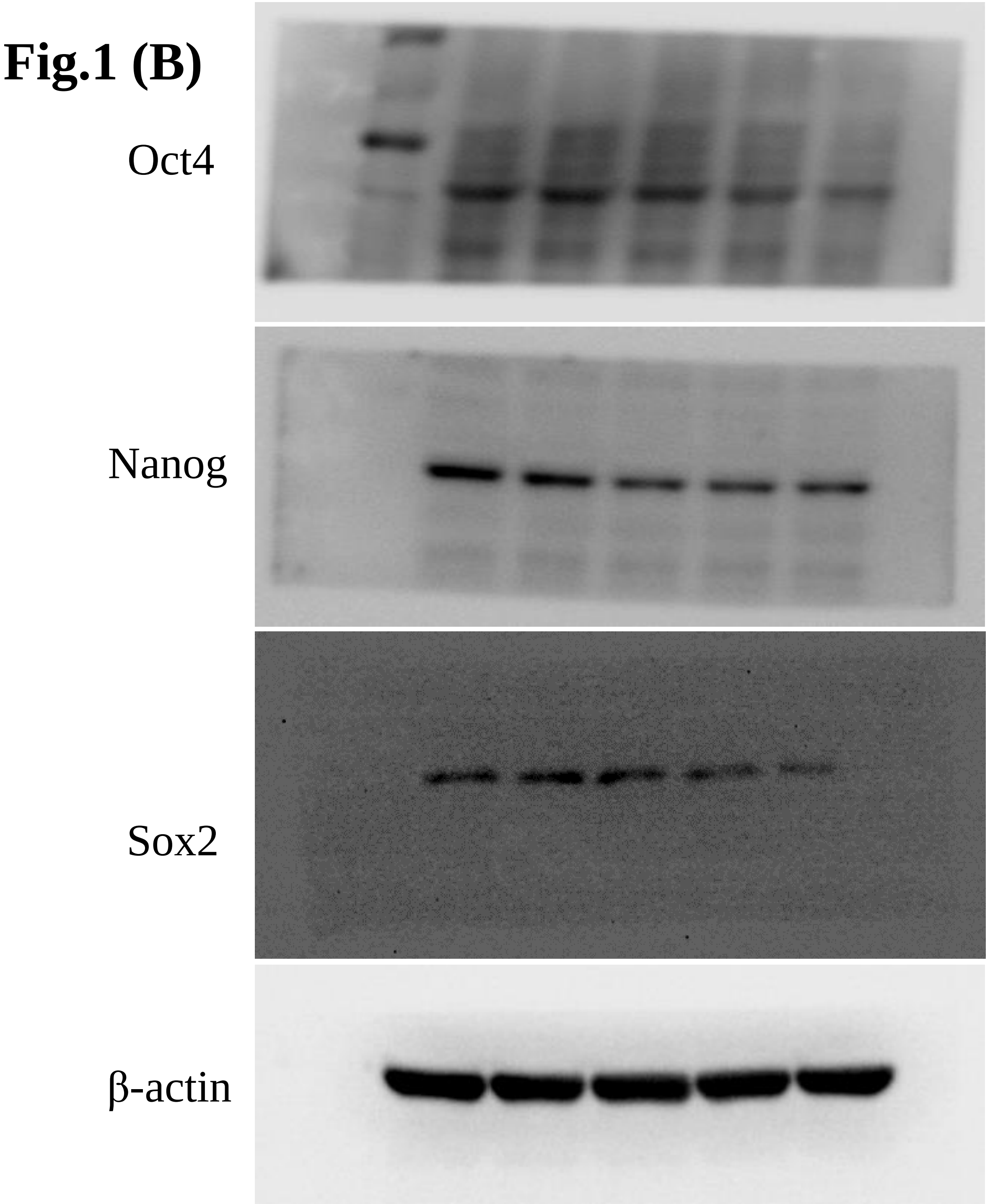
Fig.1 (B)
Oct4
Nanog
Sox2
β-actin

Fig.1 (G)

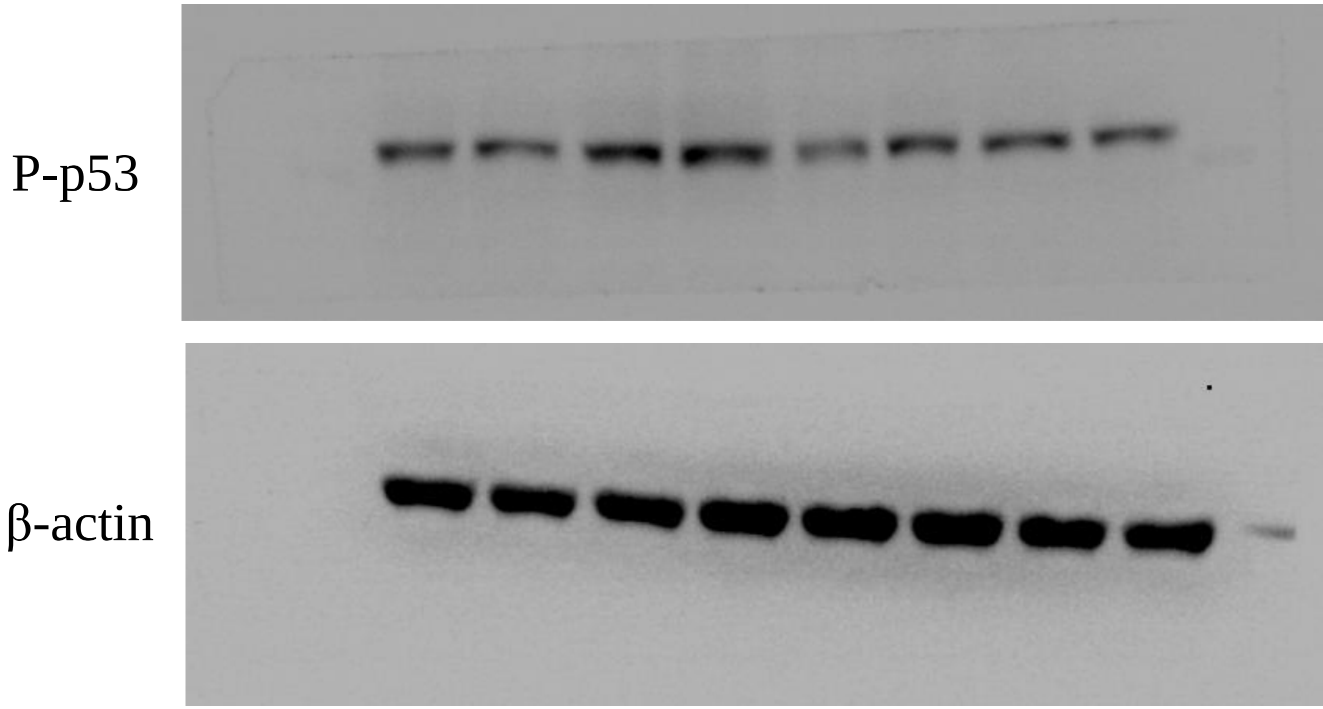

Fig.2 (A)

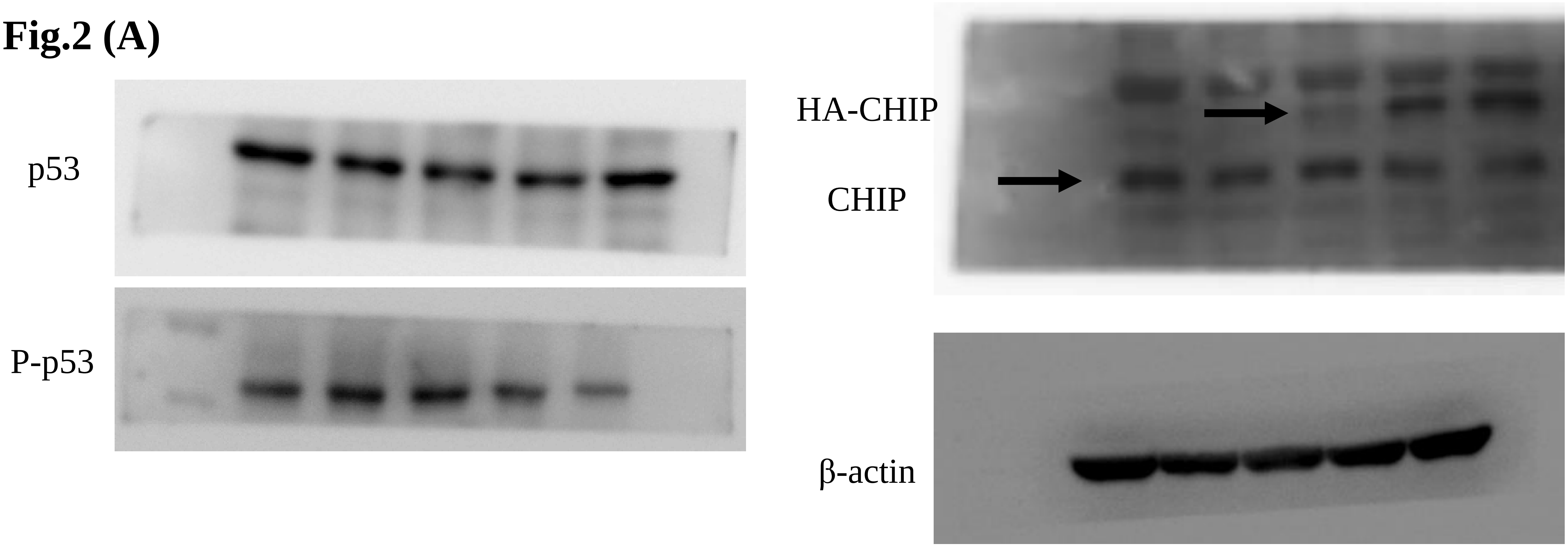

Fig.2 (B)

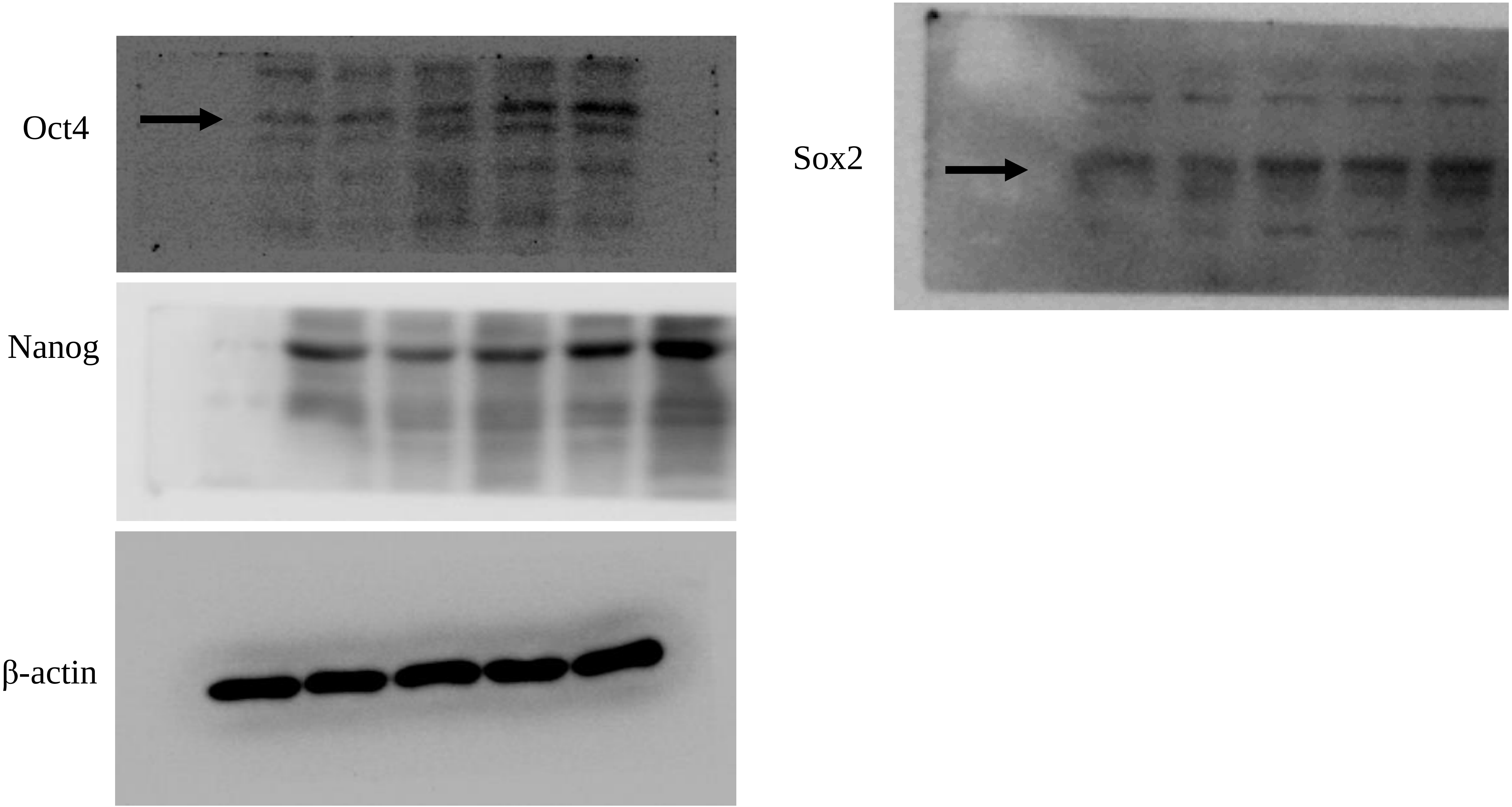

Fig.2 (C)

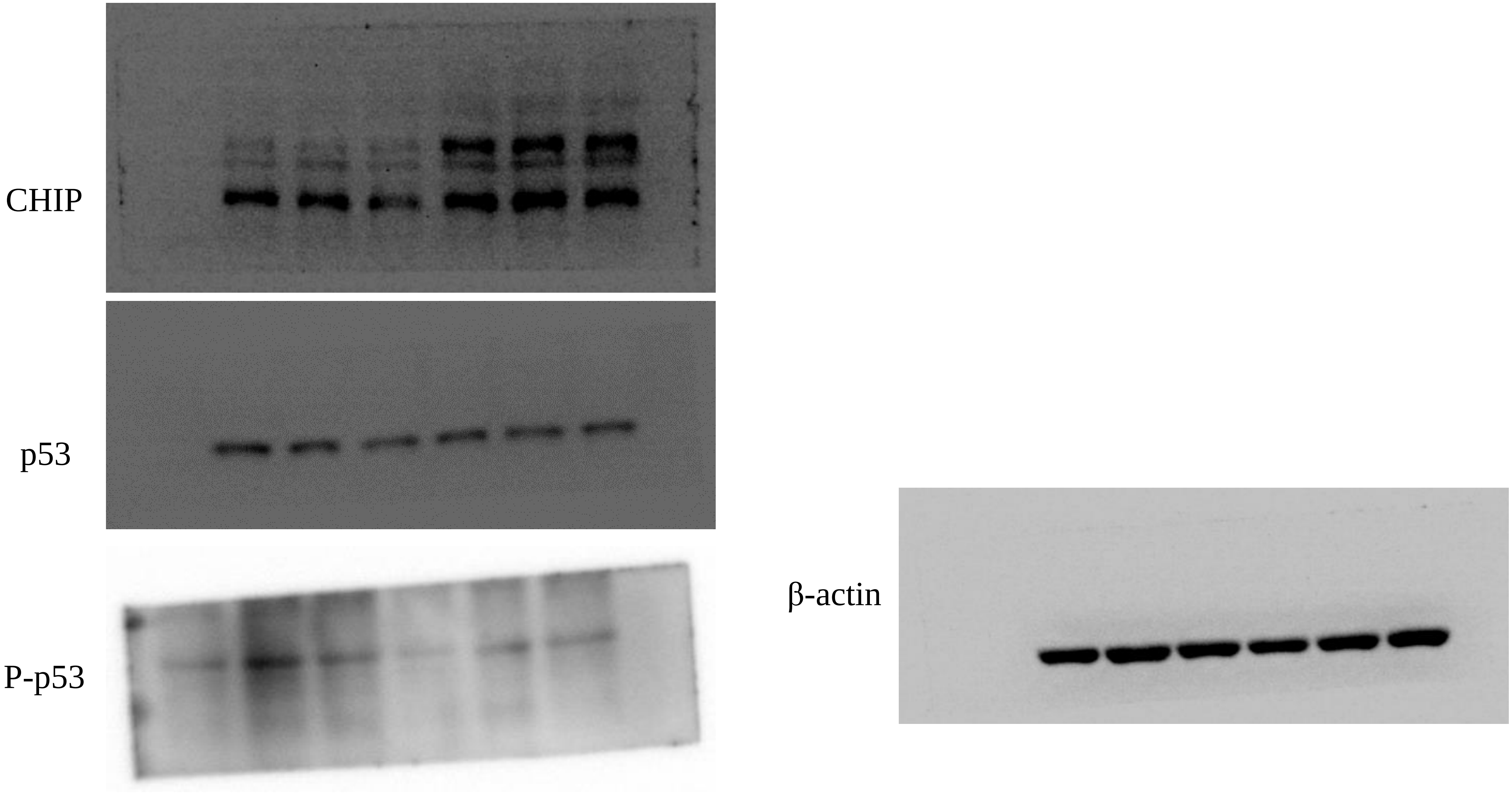

Fig.2 (D)

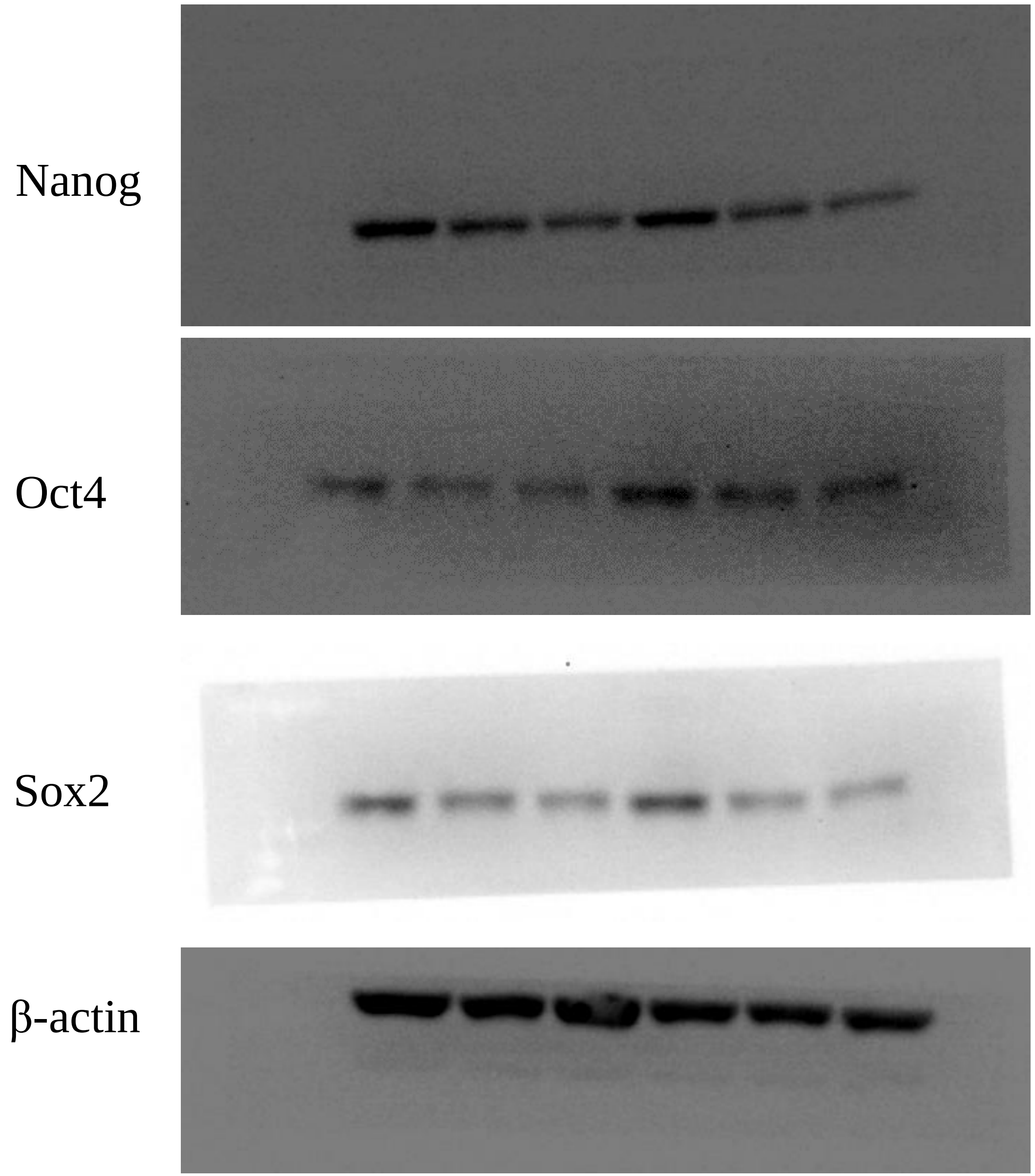

Fig.3 (A)

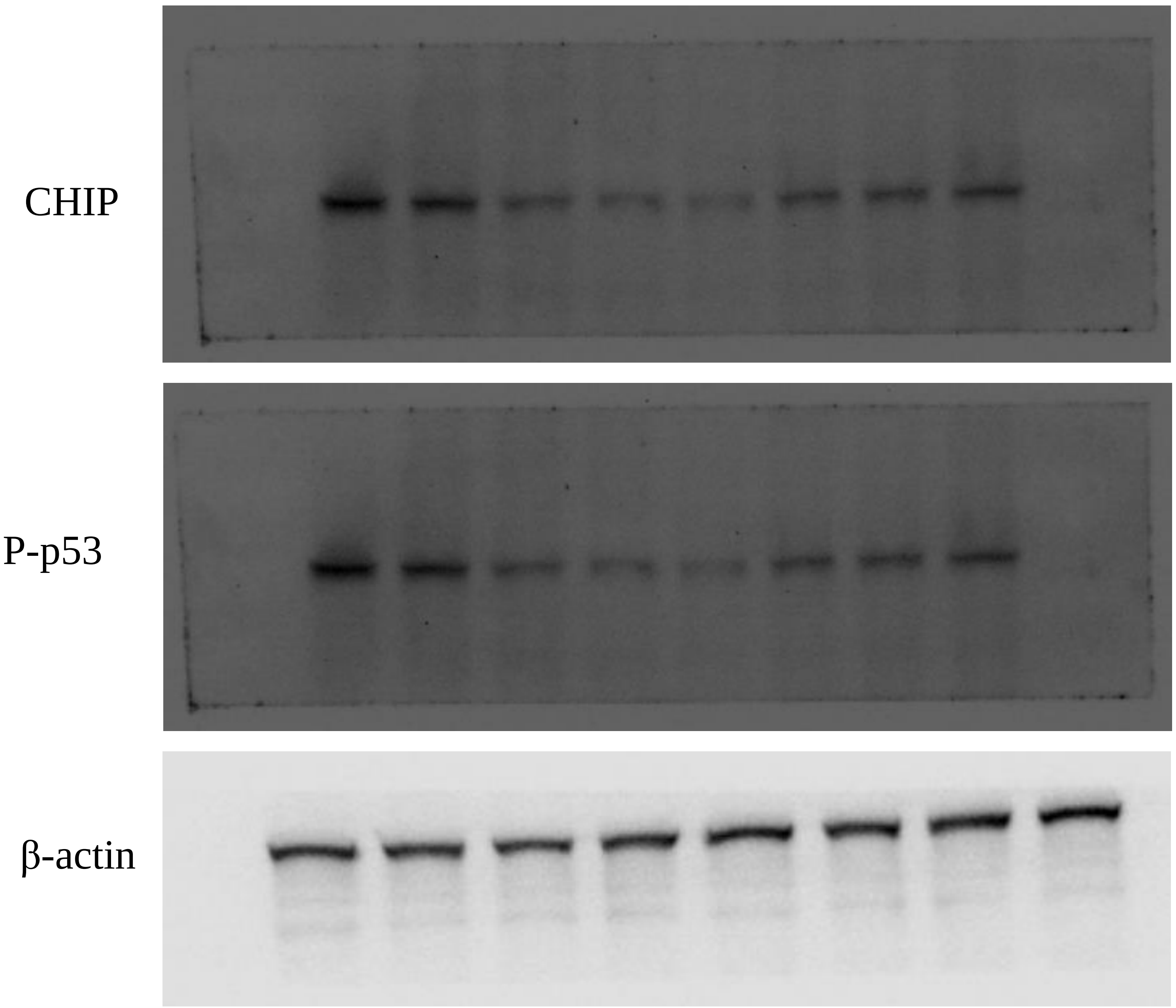

Fig.3 (B)

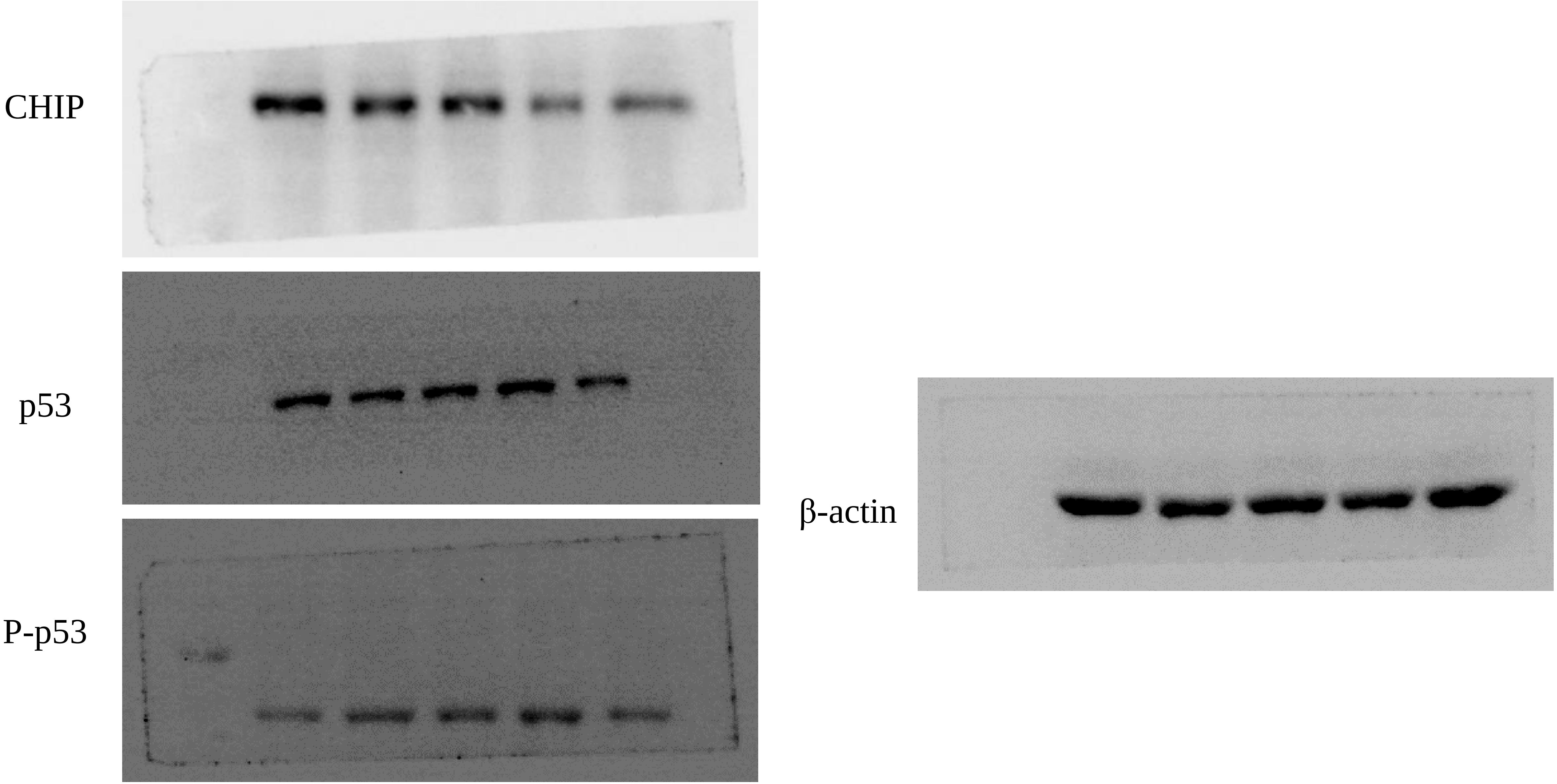

Fig.3 (C)

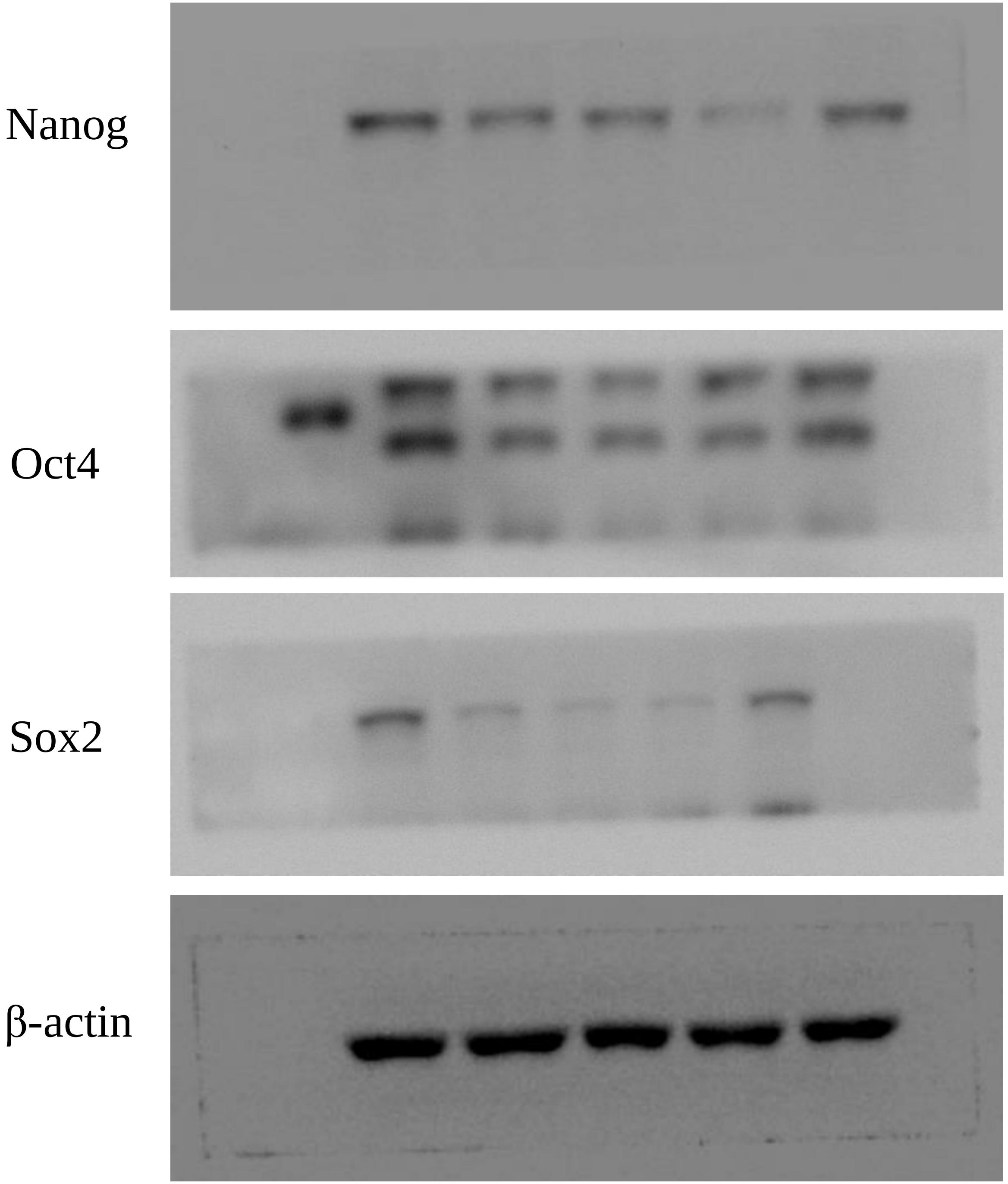

Fig.3 (D)

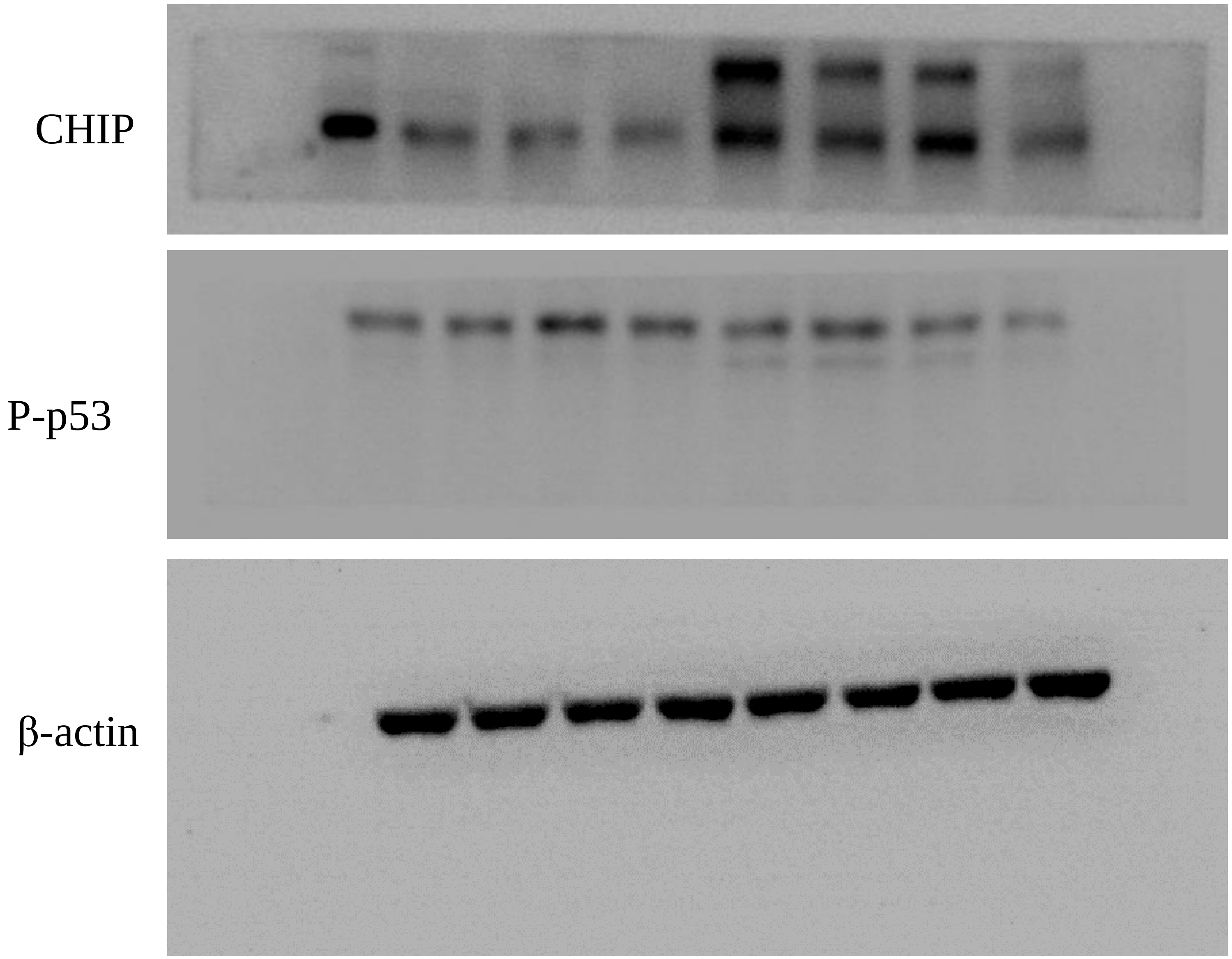

Fig.3 (E)

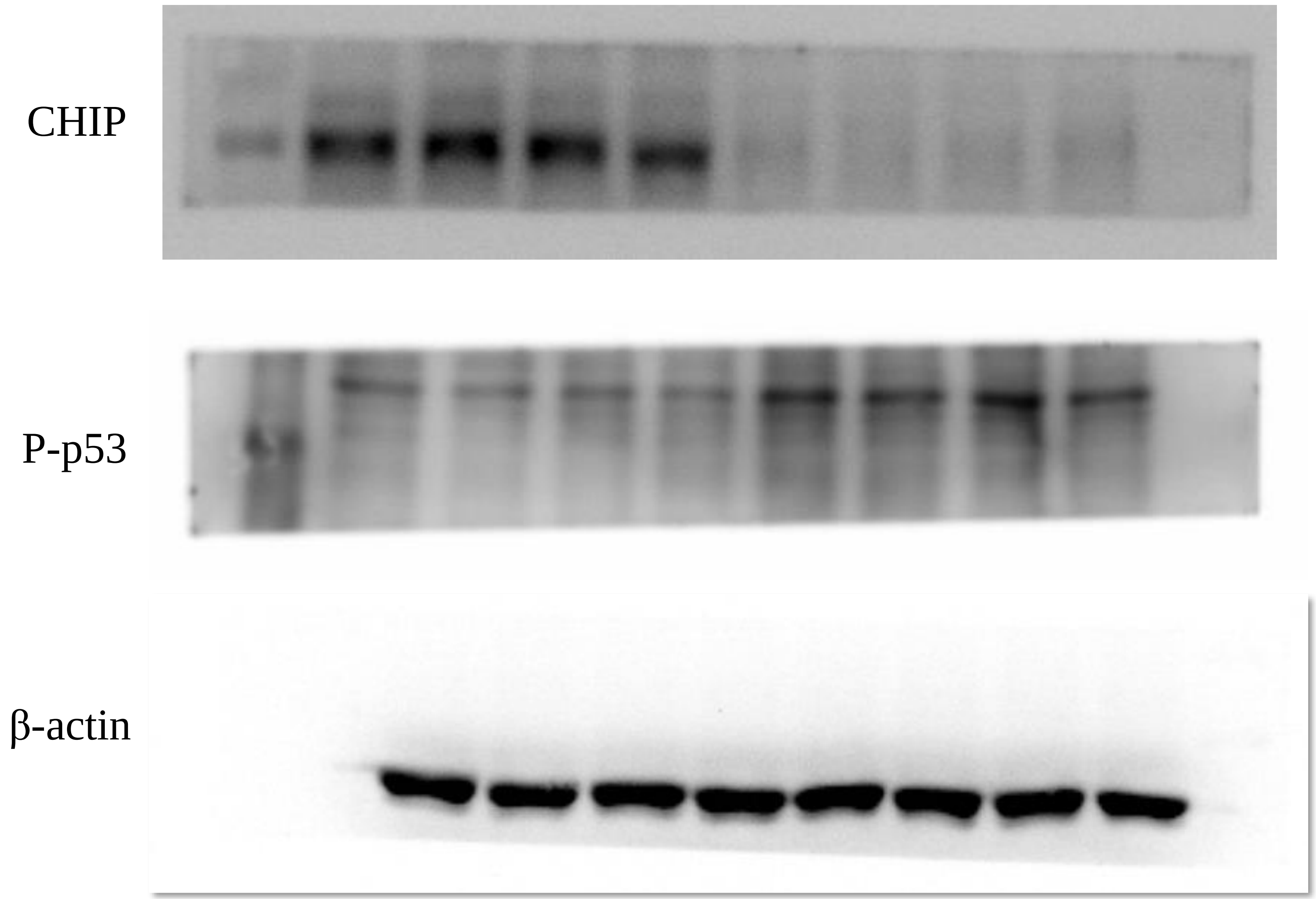

Fig.4 (A)

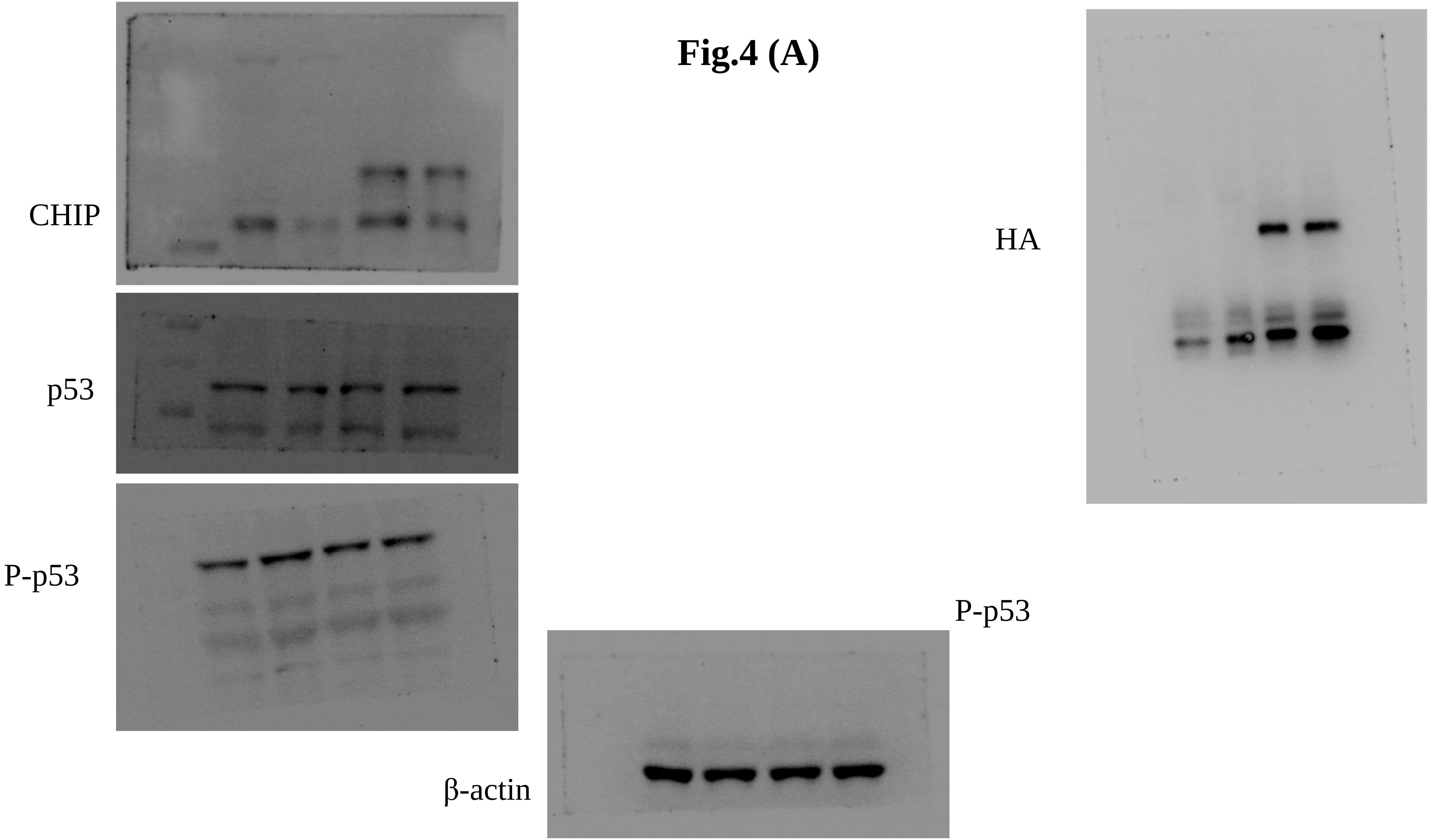

Fig.4 (B)

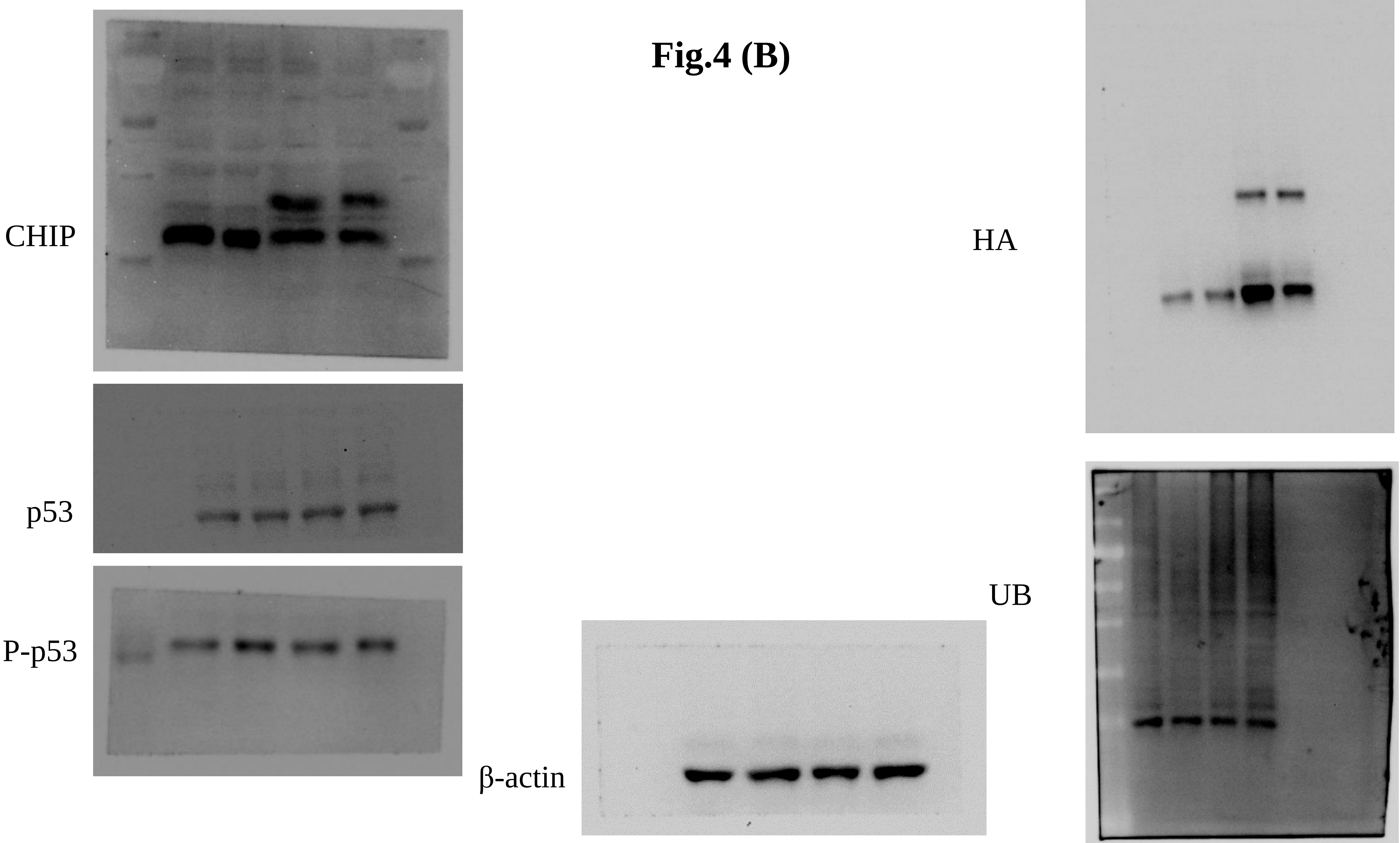

Fig.4 (C)

HA-CHIP

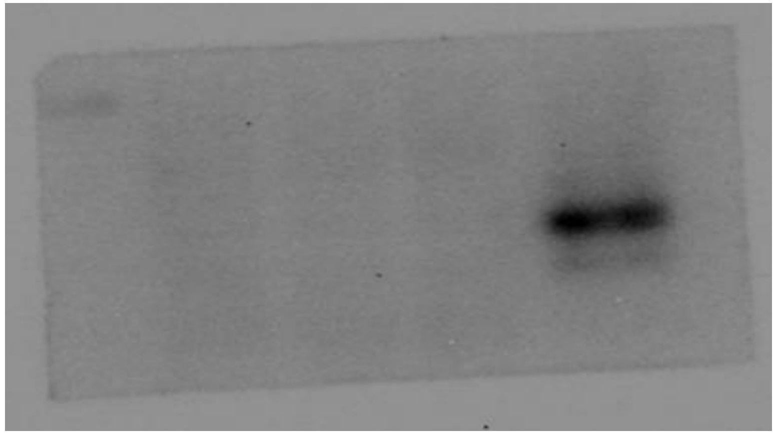

p53

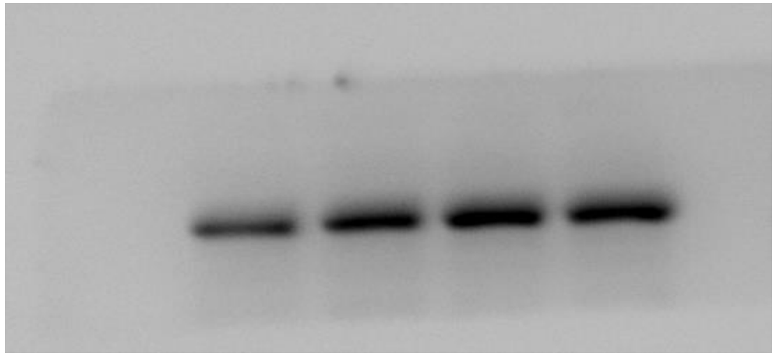

P-p53

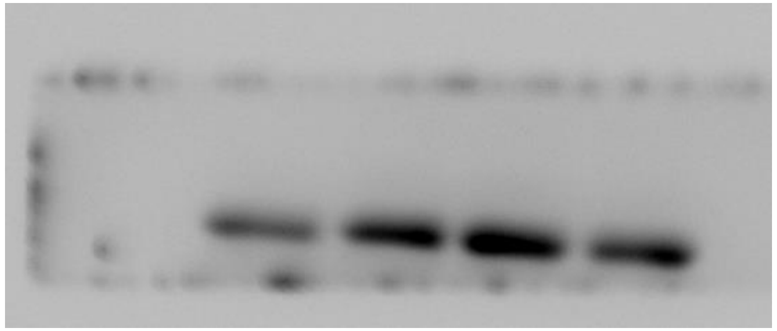

β-actin

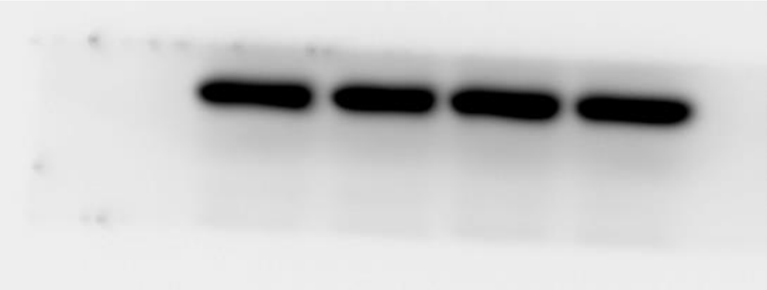

P-p53

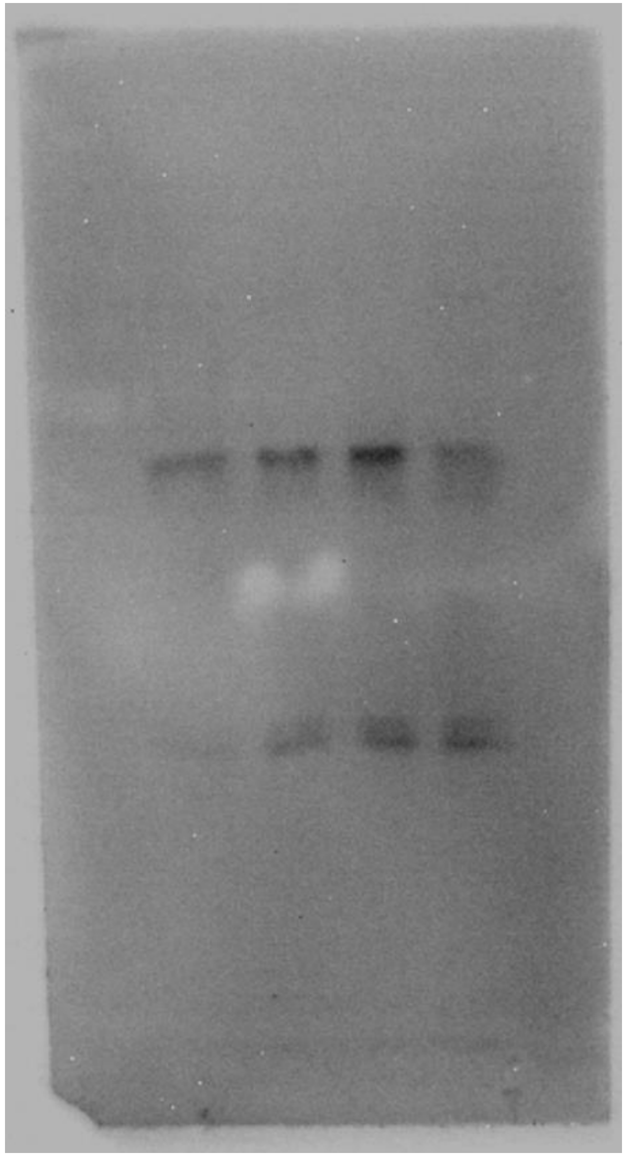

Fig.4 (D)

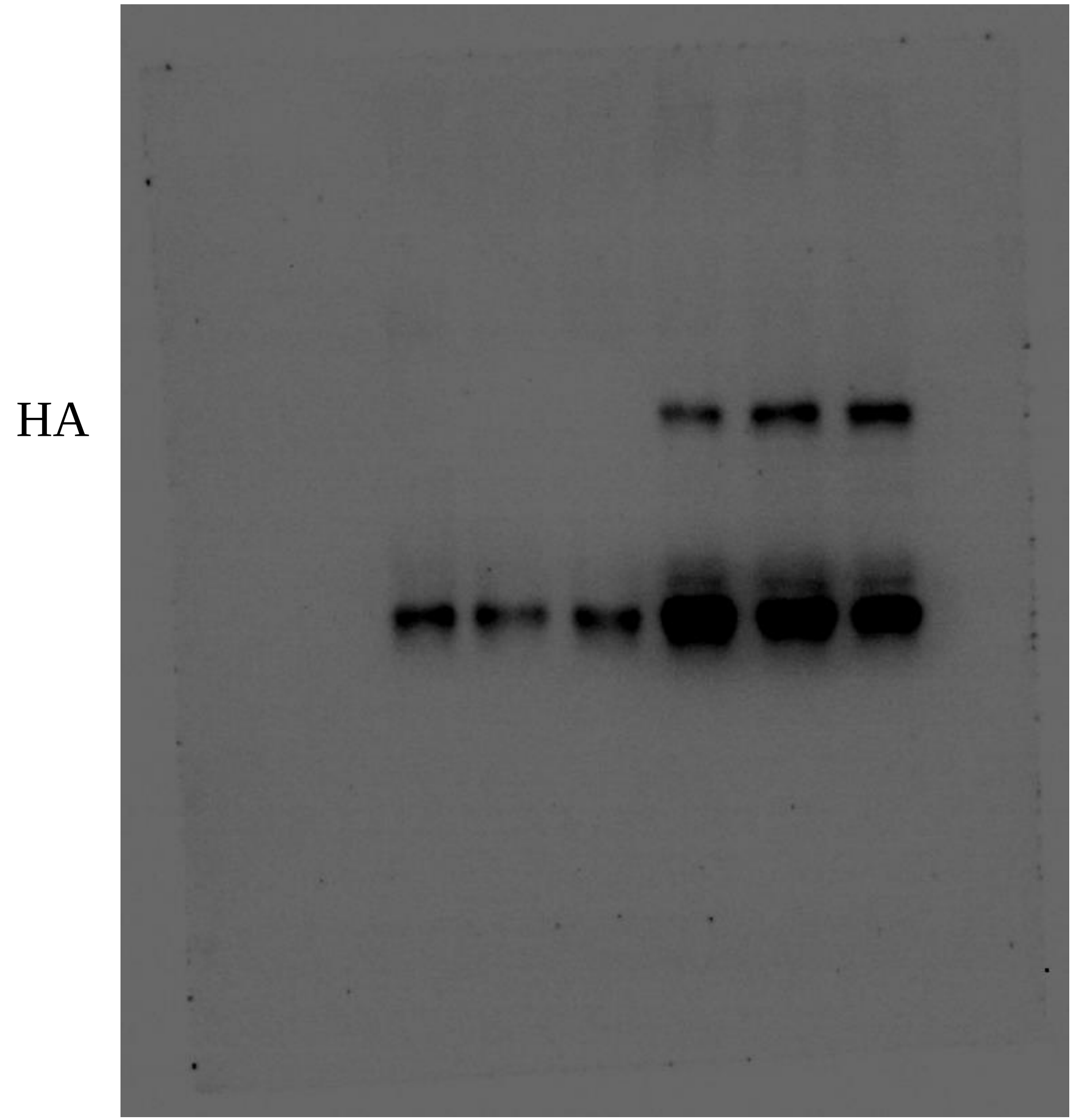

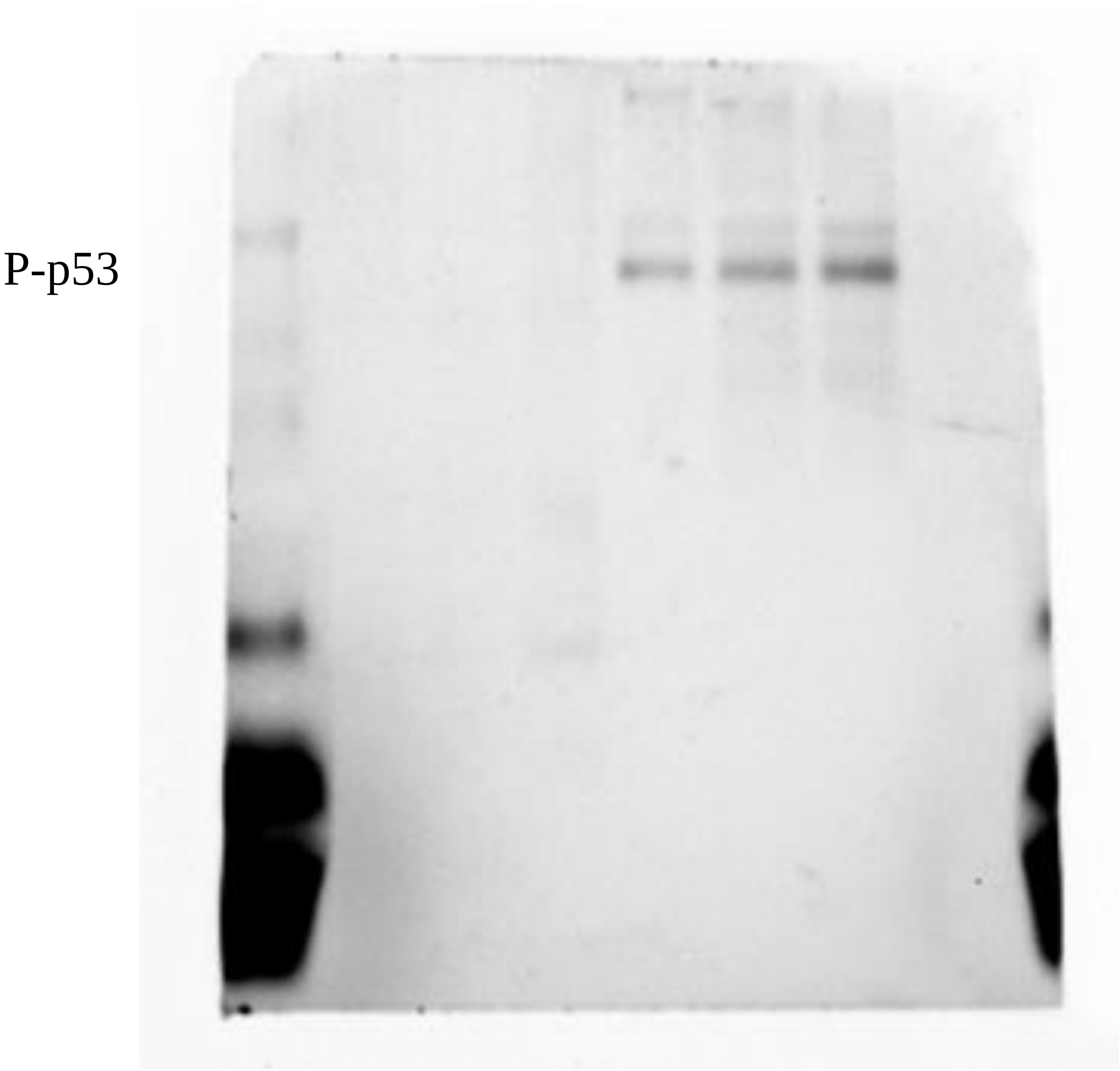

Fig.4 (D)

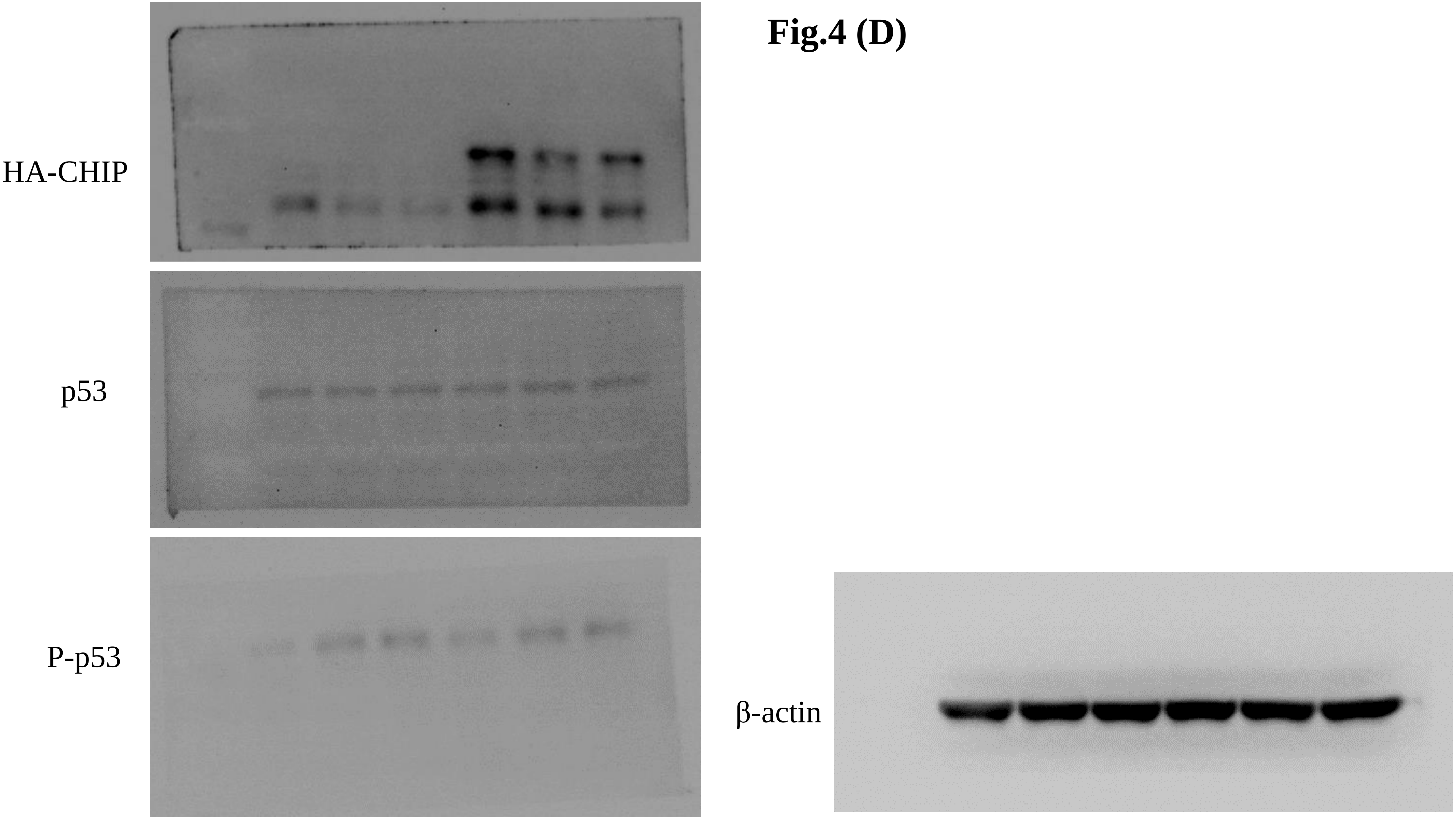

Fig.4 (E)

HA

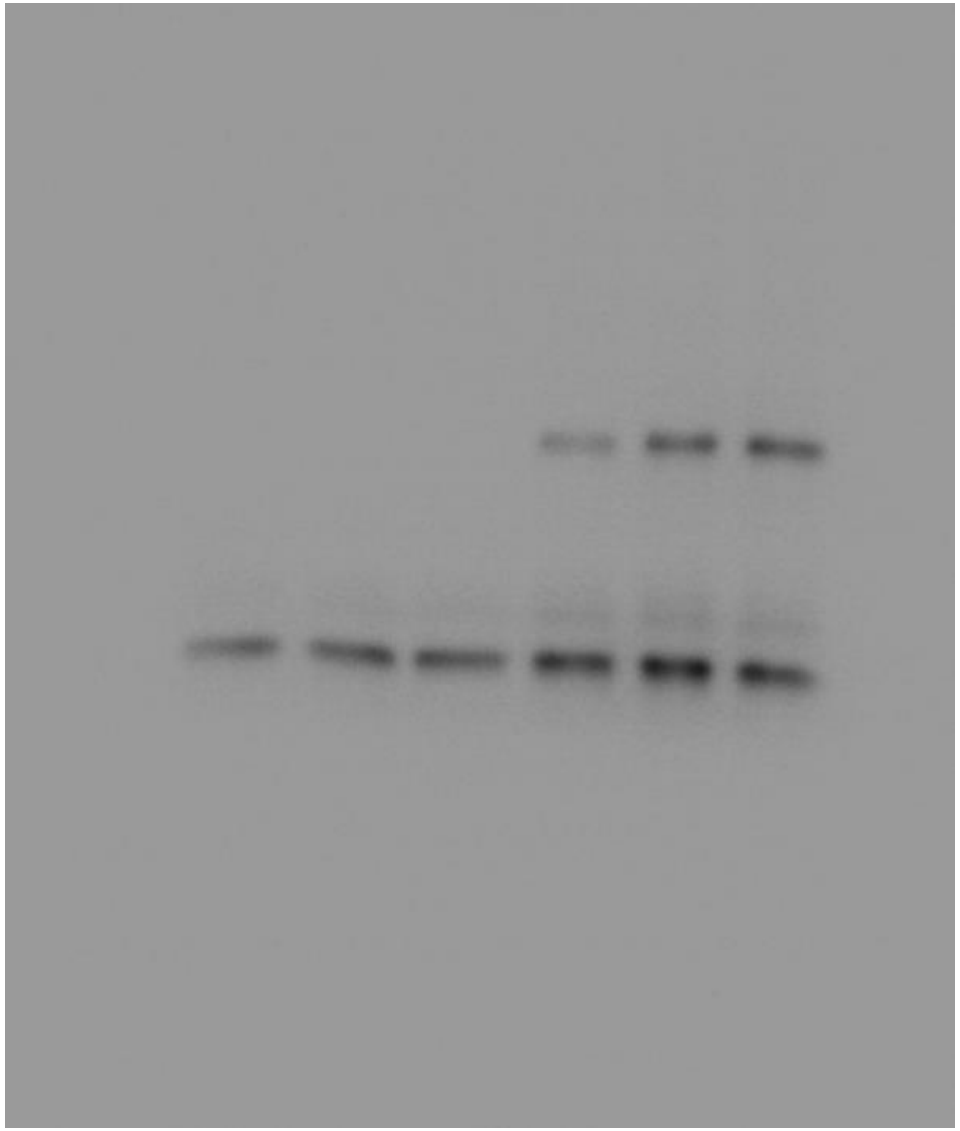

UB

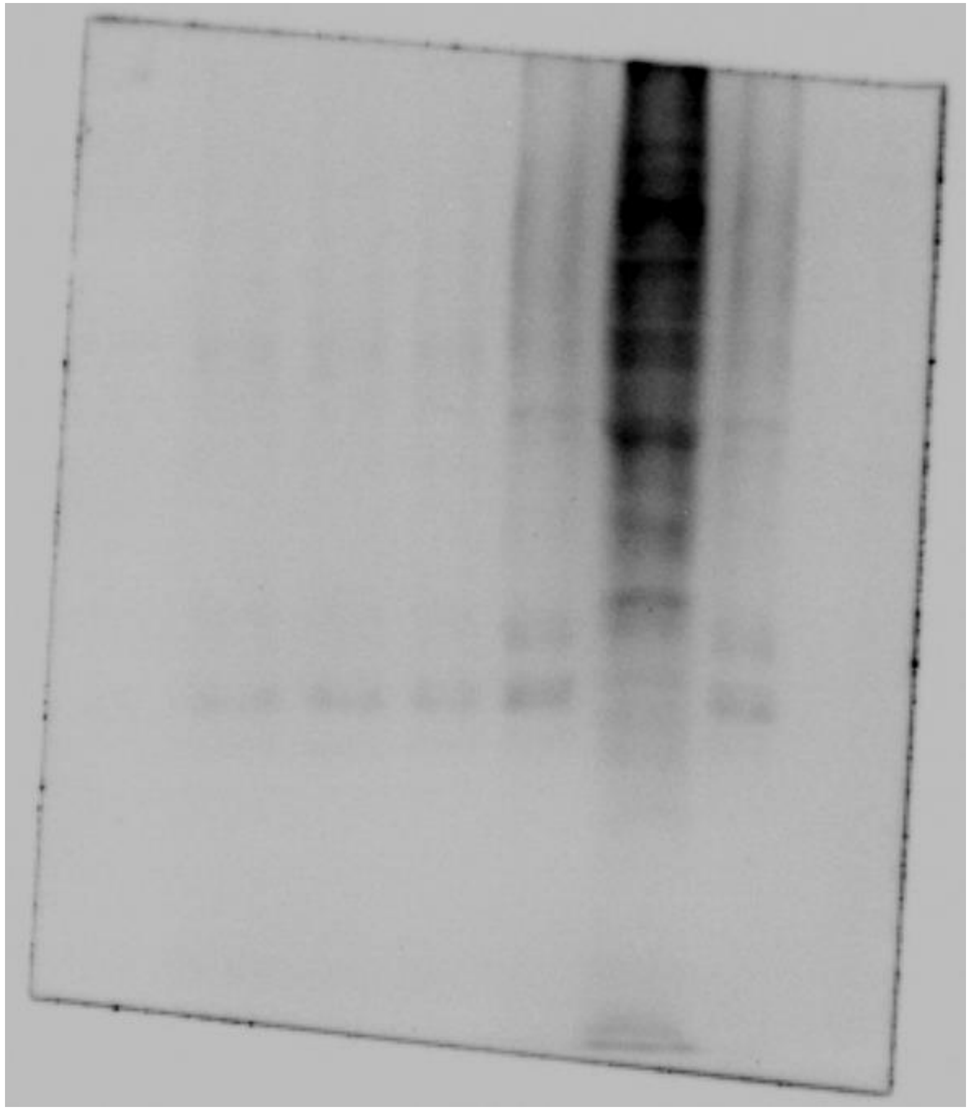

Fig.4 (E)

HA-CHIP

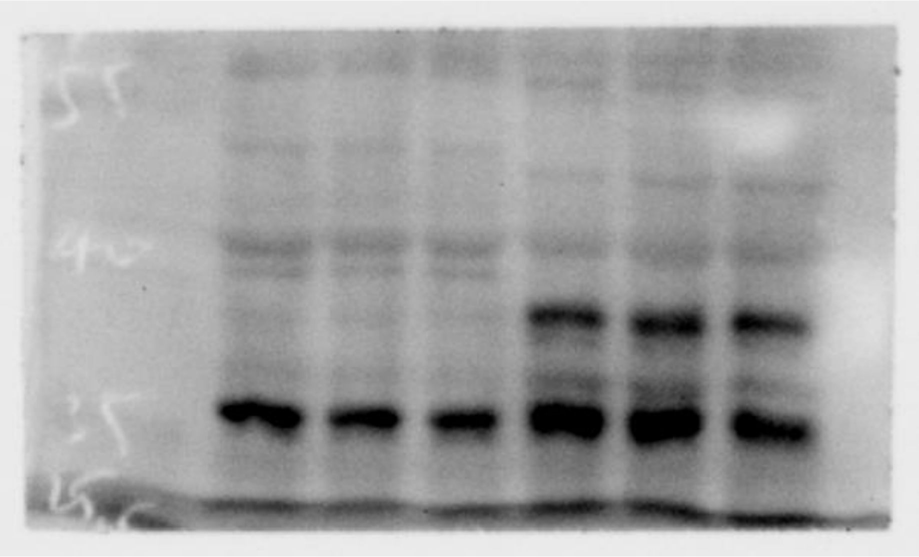

p53

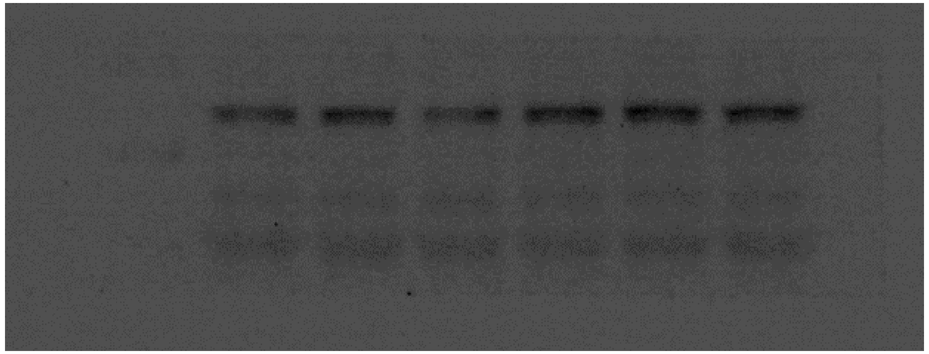

P-p53

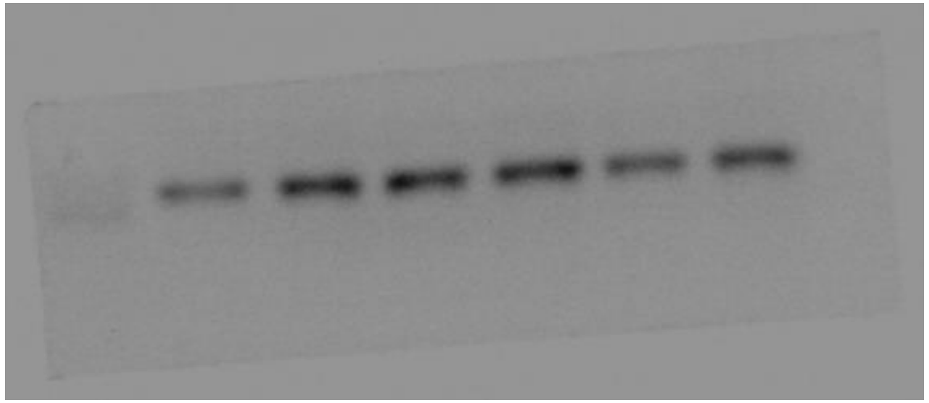

β-actin

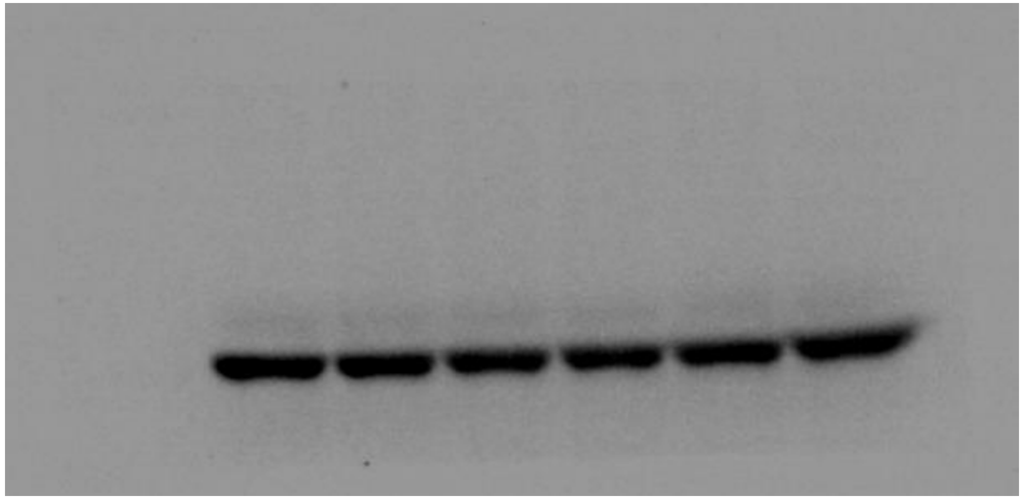

HA-CHIP

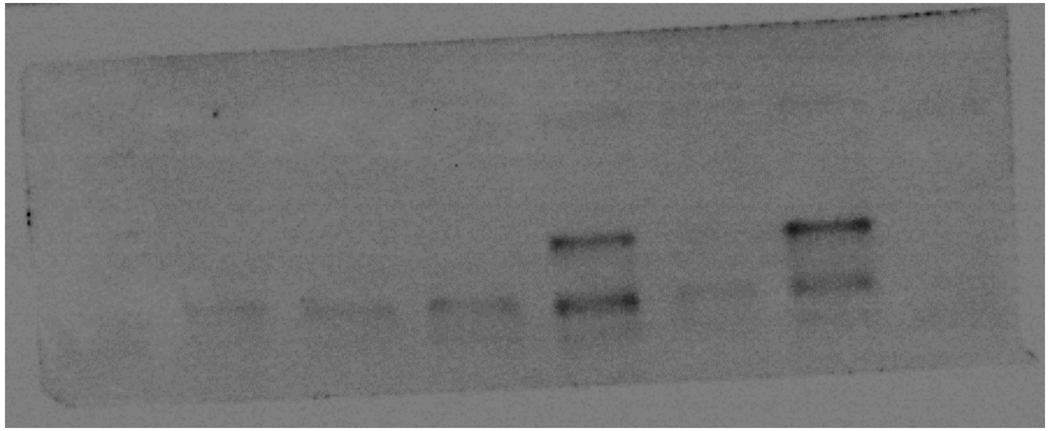

HA

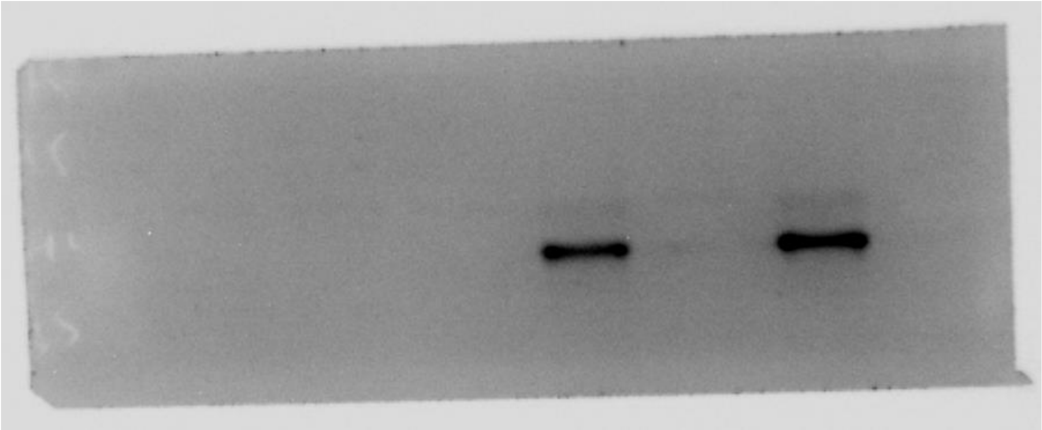

P-p53

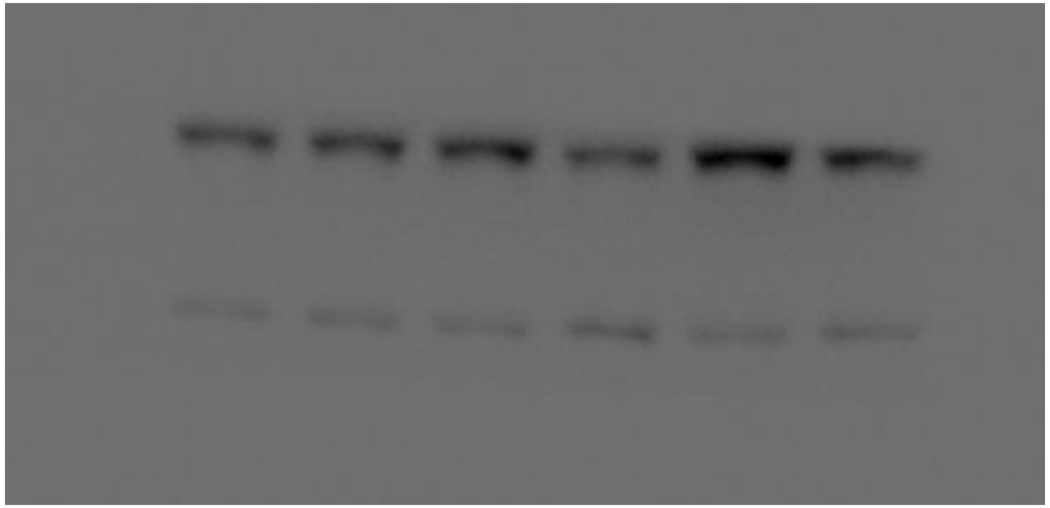

β-actin

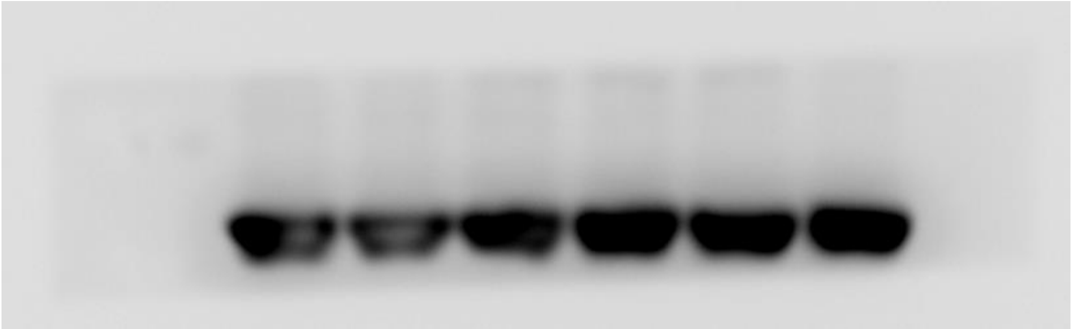

Fig.5 (A)

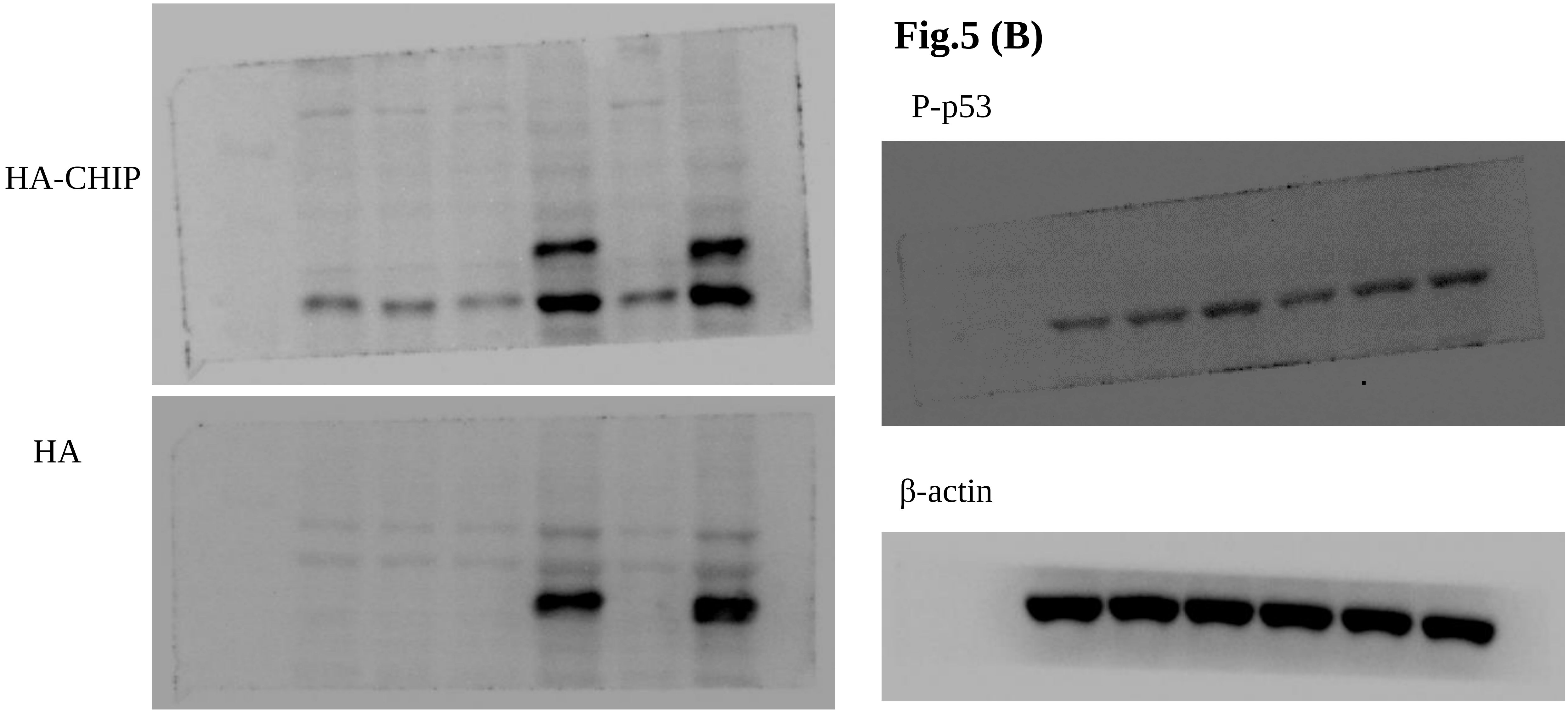

Fig.5 (B)
